# Composition and Structure of the solid electrolyte interphase on Na-Ion Anodes Revealed by Exo- and Endogenous Dynamic Nuclear Polarization—NMR Spectroscopy

**DOI:** 10.1021/jacs.4c06823

**Published:** 2024-08-22

**Authors:** Yuval Steinberg, Elias Sebti, Ilia B. Moroz, Arava Zohar, Daniel Jardón-Álvarez, Tatyana Bendikov, Ayan Maity, Raanan Carmieli, Raphaële J. Clément, Michal Leskes

**Affiliations:** † Department of Molecular Chemistry and Materials Science, Weizmann Institute of Science, Rehovot 761000, Israel; ‡ Materials Department, University of California, Santa Barbara, California 93106, United States; § Materials Research Laboratory, University of California, Santa Barbara, California 93106, United States; ∥ Department of Chemical Research Support, Weizmann Institute of Science, Rehovot 761000, Israel

## Abstract

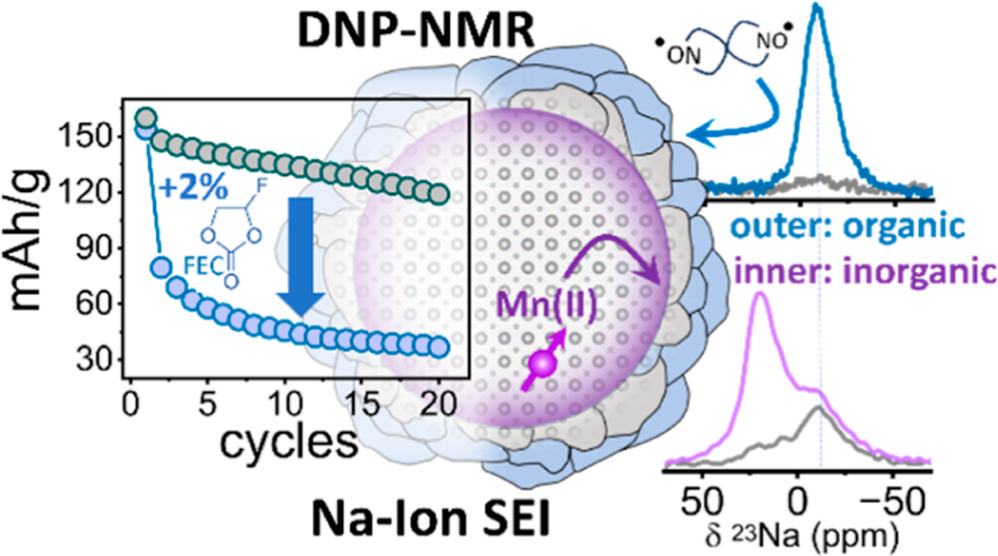

Sodium ion batteries (SIB) are among the most promising devices for large scale energy storage. Their stable and long-term performance depends on the formation of the solid electrolyte interphase (SEI), a nanosized, heterogeneous and disordered layer, formed due to degradation of the electrolyte at the anode surface. The chemical and structural properties of the SEI control the charge transfer process at the electrode–electrolyte interface, thus, there is great interest in determining these properties for understanding, and ultimately controlling, SEI functionality. However, the study of the SEI is notoriously challenging due to its heterogeneous nature and minute quantity. In this work, we present a powerful approach for probing the SEI based on solid state NMR spectroscopy with increased sensitivity from dynamic nuclear polarization (DNP). Utilizing exogenous (organic radicals) and endogenous (paramagnetic metal ion dopants) DNP sources, we obtain not only a detailed compositional map of the SEI but also, for the first time for the native SEI, determine the spatial distribution of its constituent phases. Using this approach, we perform a thorough investigation of the SEI formed on Li_4_Ti_5_O_12_ used as a SIB anode. We identify a compositional gradient, from organic phases at the electrolyte interface to inorganic phases toward the anode surface. We find that the use of fluoroethylene carbonate as an electrolyte additive leads to performance degradation which can be attributed to formation of a thicker SEI, rich in NaF and carbonates. We expect that this methodology can be extended to examine other titanate anodes and new electrolyte compositions, offering a unique tool for SEI investigations to enable the development of effective and long-lasting SIBs.

## 1 Introduction

The surge in demand for energy storage solutions in large scale systems, such as solar fields and electric vehicles, along with the rising cost of lithium, has increased the need for new and more sustainable battery chemistries. Na-ion batteries (Sodium ion batteries (SIB)) have garnered much attention as the obvious alternative to Li-ion batteries (LIB) owing to the abundance of sodium resources and its chemical similarity to lithium.^1−4^ In order to establish SIBs as a viable “post-Li” energy storage solution, electrode and electrolyte chemistries that provide high energy density and cycling stability must be developed.

Among the factors that determine the performance of a battery cell, the formation of a solid electrolyte interphase (SEI) is essential for the cell’s cycling stability. The SEI is a nanosized heterogeneous solid layer that forms at the anode-electrolyte interface due to decomposition of the electrolyte constituents, and is comprised of various organic and inorganic phases.^5−7^ A beneficial SEI prevents further degradation of the electrolyte by blocking electron transfer and passivating the interface, but still allows for fast ionic transport between the anode and the electrolyte. Conversely, a detrimental SEI will cause rapid consumption of the electrolyte, large irreversible capacity and poor cycling stability. The effectiveness of the SEI in stabilizing cell cycling is dictated by its composition and structure. As a result, characterizing these SEI properties is of utmost importance for the development of any new battery material.^5,7−9^ In the past decades, the SEI has been studied in depth in the context of Li anodes, with several models proposed for the SEI architecture. The common notion is that the SEI is comprised of organic species in the parts close to the electrolyte, and a more stable inorganic layer closer to the interface with the anode material.^10,11^ While the study of the SEI in LIBs is quite comprehensive and a general idea of its composition and structure is established, this is not the case for SIBs. Therefore, there is a dire need to understand the properties of the SEI forming on Na anodes in order to identify paths toward SEI stabilization.^12−14^

Due to the nanometer-scale thickness, heterogeneity, and disordered nature of the SEI, its characterization using conventional materials science tools such as X-ray diffraction (XRD) or electron microscopy (EM) is often limited. One of the most prominent tools used in SEI research is X-ray photoelectron spectroscopy (XPS), which enables the detection of interfacial phases (interphases) to determine the SEI composition as well as its structure through depth profiling using ion sputtering.^12,15,16^ However, the chemical resolution of XPS is often limited and radiation damage may alter the composition of organic SEI components. Solid state NMR (ssNMR) spectroscopy provides excellent chemical resolution for many of the elements in the periodic table and, as such, has been used extensively in the past few years to study both bulk^17−19^ and interfacial transformations^20,21^ that occur during electrochemical cycling of various electrode materials. However, the study of the SEI by ssNMR is limited by its intrinsically low detection sensitivity, leading to prohibitively long measurement times or the inability to detect phases containing elements with NMR active isotopes that have low natural abundance and/or low gyromagnetic ratio (such as ^13^C, ^15^N, ^17^O, ^33^S). Furthermore, the low sensitivity prevents acquiring correlation experiments which can provide critical information regarding the SEI’s structure. Isotope enrichment of the electrolyte components has been employed to circumvent this limitation in the case of ^13^C detection,^22,23^ yet is not a general solution for all electrolyte compositions and isotopes.

Magic angle spinning-dynamic nuclear polarization (MAS-DNP) offers an alternative and efficient approach to address sensitivity limitations in ssNMR.^24−26^ In DNP, the high polarization of unpaired electrons is transferred to nearby coupled nuclei by irradiating the sample with microwaves. In materials science applications, DNP is typically performed by wetting the sample of interest with a solution of stable nitroxide biradicals, resulting in increased surface sensitivity—a technique called DNP-surface enhanced NMR spectroscopy (DNP-SENS).^25,27^ Developments in the synthesis of nitroxide biradicals as polarizing agents has enabled signal enhancements in excess of 200, allowing for the detection of surface species via NMR of active nuclei with low abundance such as ^17^O and ^13^C.^28−33^ This exogenous DNP approach has been successfully used to identify the organic phases making the external layers of the SEI formed on reduced graphene oxide and Si anodes.^34,35^ However, the information gained by exogenous DNP was shown to be limited to the outer layers of the SEI, and does not enable detection of the entire SEI composition. An alternative approach for DNP is to utilize endogenous sources of polarization, such as conduction electrons in the case of conductive electrodes^36^ or paramagnetic metal ions introduced as dopants in the case of ceramic electrodes.^37^ Metal ions DNP (MIDNP) has been successful in enhancing the signal originating from the bulk of inorganic solids, enabling detection of low sensitivity nuclei such as ^17^O and ^89^Y.^38−42^ Recently, we have shown that with MIDNP the polarization from dopants introduced in the particle’s bulk can extend to its surface, providing sensitivity in the detection of 2–5 nm thick coating layers.^43^ These results highlight one of the benefits of MIDNP, which enables detection of buried solid interfaces that are not accessible to exogenous sources of polarization. However, to date, MIDNP has not been applied to study the native, electrochemically formed SEI.

Here we provide an in-depth study of the SEI composition and structure formed on SIB anodes in different electrolytes. As yet, most studies of the SEI on SIB anodes have focused on Na metal batteries or hard carbon (HC),^44−46^ both of which offer high capacity and low operation potential. However, their low potentials also leads to nonuniform Na deposition at their surface and is thus a safety concern.^47−49^ Among the alternatives considered for SIB applications, titanium oxide-based anodes are advantageous as they are low toxicity materials with abundant constituent elements, can be obtained through low-cost synthesis methods, and exhibit reasonably low sodiation potentials, while also preventing Na plating during fast cycling.^50^ Li_4_Ti_5_O_12_ (LTO) is a known anode material used for LIBs, and in the past decade it has also gained attention as a possible SIB intercalation material.^51,52^ While the intercalation mechanism has been thoroughly investigated, little is known about the SEI formed on titanate anodes.

It is well established that the electrolyte composition dictates the SEI composition. As such, one approach to control the SEI content is the use of sacrificial additives in the electrolyte, which decompose prior to the electrolyte, ideally resulting in a stable passivating SEI. Vinylene carbonate and fluoroethylene carbonate (FEC) are common additives used in LIBs, which have been shown to have beneficial effect on the SEI in certain electrode–electrolyte combinations.^7,53−56^ FEC has also been investigated for SIB cells,^57,58^ and was found to have a beneficial effect on the SEI formed on HC, mainly attributed to the formation of NaF. In contrast, Palacín et al. found that adding 2% FEC to their battery electrolyte had a detrimental effect for HC anodes.^59^ Dahbi and co-workers also found that addition of FEC can worsen the performance of the battery when carboxymethyl cellulose is used as a binder, instead of the common polyvinyl difluoride (PVDF).^60^ While it is generally accepted that the use of FEC results in the formation of NaF, its effect on the SEI is not always beneficial. In particular, the function of NaF as an ion-conducting medium for Na ions is questionable, based on density functional theory (DFT) calculations.^61^ Thus, the role of additives such as FEC on SIB performance remains an open question.

**Scheme 1:**
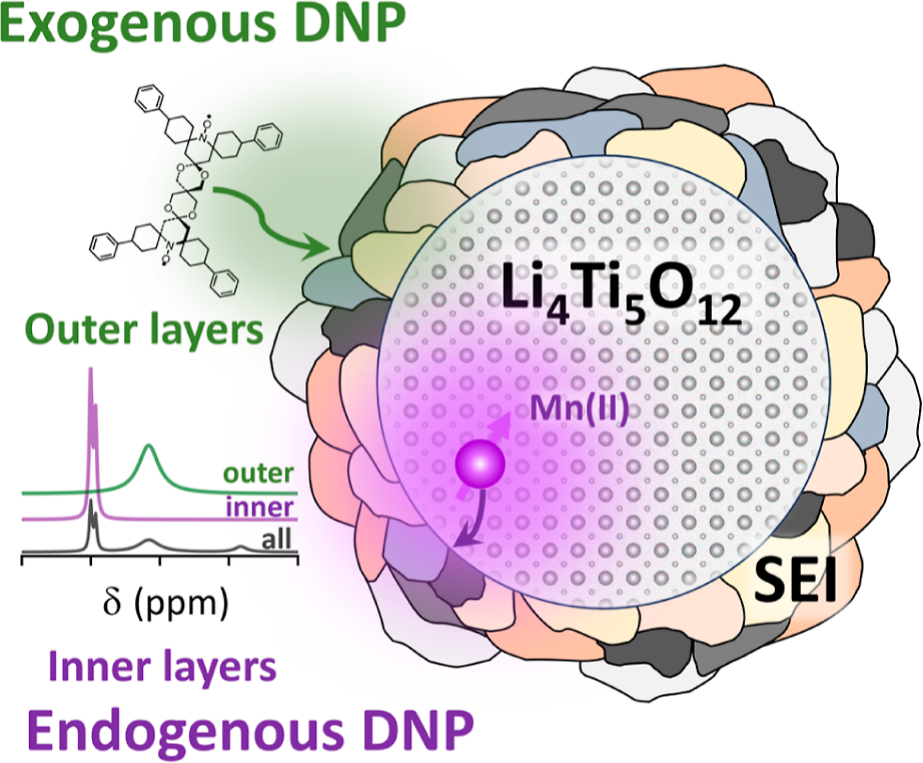
Proposed Approach for Analyzing the Native SEI Formed on LTO Particles: Endogenous DNP with Mn(II) Metal Ions Enhances the Inner SEI Layer (Purple) whereas Exogenous DNP with TEKPol Biradicals Enhances the Outer SEI Surface (Green) The different phases making the SEI are represented by the different colored shapes on the LTO surface.

In order to understand the interplay between electrolyte composition, SEI formation, and its functionality as an ion conductor, there is a need for nondestructive and sensitive characterization tools. Here, we describe and employ a powerful ssNMR—DNP combination for determining both the SEI composition and its structure. Multinuclear ssNMR spectroscopy and DNP are used to obtain a detailed compositional map of the SEI formed on LTO used as SIB anode. Exogenous (from nitroxide biradicals) and endogenous (from Mn(II) dopants) polarization sources are used to nondestructively determine the architecture of the native SEI. Scheme 1 shows a representation of an heterogeneous SEI, made of different phases (represented by the different colors), and the two DNP approaches employed in this work, using exogenous and endogenous polarization sources to highlight different parts of the SEI.

We first describe the optimization process for LTO synthesis, with respect to its electrochemical performance in SIBs and address the challenges in applying exogenous and endogenous DNP to cycled electrodes. Next, we discuss the effect of the FEC additive on the electrochemical performance of LTO batteries. We then provide a detailed analysis of the SEI composition from spectral assignment of ^23^Na, ^19^F and ^13^C ssNMR resonances to specific SEI phases. Quantitative NMR measurements and double resonance experiments, enabled by sensitivity enhancements from DNP and supported by DFT calculations of NMR parameters, are used to identify the process of Na–Li exchange. We then determine the architecture of the native SEI by exogenous and endogenous DNP, identifying changes in the LTO-SEI interface and subsurface layers upon irreversible sodiation. The results are discussed in the context of XPS depth-profiling experiments. Finally, we propose a layered model for the SEI formed on LTO anodes based on the compositional information gained from ssNMR and the structural insights obtained by enhancing the ssNMR signal via exogenous and endogenous DNP. The presented approach provides a novel framework for examining new electrolyte compositions in search of stable, ionically permeable SEIs for titanate-based SIBs. Furthermore, we expect the DNP-NMR methodology developed herein to benefit the study of the SEI in a variety of titanate oxides used as anodes in Li, Na and K batteries.

## 2 Materials and Methods

### 2.1 Materials

Li_4_Ti_5_O_12_ (LTO) was synthesized based on the hydrothermal procedure by Wan et al.^62^ 168 mg of LiOH·H_2_O (Sigma-Aldrich, >99%) was dissolved in 17 mL of high-performance liquid chromatography grade ethanol (J.T. Baker). 1.7 mL of titanium(IV) *n*-butoxide (Alfa Aesar, >99%) was added dropwise to the solution, resulting in a white suspension that was stirred for 24 h in a closed vial to avoid evaporation and moisture. The ratio of precursor was stoichiometric according to the LTO formula. Mn(II) doped LTO was synthesized by adding appropriate amounts of Mn(NO_3_)_2_ (Alfa Aesar, 99.99%) after adding the titanium(IV) *n*-butoxide. The suspension was then transferred to a 125 mL Teflon-lined reactor (Parr) where 21.25 mL of deionized water were added and stirred for 30 min. The reactor was placed in an oven at 180 °C for 36 h. The resulting gel was washed 3 times with ethanol and left to dry in an oven under air at 80 °C for 12 h. After drying the gel, a powder was obtained containing noncrystalline oxides. Finally, the powder was calcined at 700 °C for 2 h in a tube furnace under flow of a reductive gas mixture containing H_2_/N_2_ (5:95). The calcined LTO was kept in an argon filled glovebox.

### 2.2 Materials Characterization

The phase purity of the LTO powders was determined by powder XRD on a TTRAX-III Rigaku diffractometer operating at 50 kV and 200 mA, using a rotating Cu anode. XRD measurements were performed by scanning the angle between 10 and 120° at a rate of 1°/min. The XRD results were analyzed using JADE 2010 software.

EM images were taken using a Sigma-Zeiss scanning electron microscope (SEM) operating at 5 kV. Scanning transmission electron microscopy high-angle annular dark-field (STEM-HAADF) together with energy dispersive X-ray analyses were carried out using the Talos F200X G2 model. The STEM samples were prepared on a lacey carbon 400 mesh copper grid. The sample preparation took place inside an argon-filled glovebox, where a suspension of the sample in dimethyl carbonate (DMC) solvent was carefully drop-cast onto the grids. Following this, the samples were subjected to vacuum drying to ensure complete removal of any residual solvents. To prevent air exposure, the samples were transported to the facility in an argon-filled airlock box and quickly transferred to the instrument.

Continuous wave (CW) EPR measurements were performed using a Q-band (35 GHz) Bruker ELEXYS E-580 spectrometer fitted with a Q-band resonator (EN5107-D2). The temperature was controlled by a Bruker FlexLine cryogen free VT system ER4118HV-CF5-H, and experiments were performed at room temperature and 100 K. CW X-band (9.4 GHz) EPR spectra were collected on a Bruker Magnettech ESR5000 spectrometer with a modulation frequency of 100 kHz.

### 2.3 Electrochemistry

Coin cells (type 2032, TOB) were assembled in an Ar-filled glovebox in half cell configuration with Na metal (Sigma-Aldrich) and LTO as the two electrodes. For the characterization of the SEI formed on LTO, high loading of active material (9–10 mg) was necessary. To this end, the LTO electrodes were made by placing the LTO powder on top of 13 mm Al foil used as the current collector. The LTO powder was then pressed using a manual press at 5 tons for 3 min, resulting in a flat and homogeneous layer of LTO on top of the Al current collector, labeled pressed powder of LTO (ppLTO, pressed powder LTO). No binder or carbon black were used in these electrodes, for reasons that will be discussed later on. In addition, conventional electrode films were prepared by mixing together LTO, carbon black (C-45) and PVDF binder (in weight ratio of 70:20:10, respectively). *N*-methylpyrrolidone (NMP) was added to the mixture and stirred overnight, then the resulting slurry was spread on top of Al foil using a 100 μm doctor blade. The film was then dried overnight in a vacuum oven at 100 °C and 13 mm electrodes were cut using a punch, then pressed with a manual press at 5 tons for 3 min. Both the ppLTO and conventional film batteries were placed in a vacuum oven at 100 °C overnight before placing them in the glovebox. A borosilicate separator (Whatman, Sigma-Aldrich) was used between the two electrodes and was soaked with 8 drops of the electrolyte, 1 M sodium bis(fluorosulfonyl)imide (NaFSI) in ethylene carbonate (EC) and diethyl carbonate (DEC) in a 1:1 ratio (Solvionic). Currents for C-rates were calculated from the specific capacity of LTO, 175 mAh/g. Galvanostatic measurements were performed at room temperature using a BCS-805 battery cycler and Bio-Logic VMP3 cycler (Biologic Science Instruments) in a potential window of 0.5–3.0 V at a rate of *C*/10.

### 2.4 NMR and DNP Sample Preparation

Following cycling, the battery cells were disassembled inside the glovebox to prevent contamination of the SEI. The cells were opened and the ppLTO electrodes were gently scraped off using a blunt plastic spatula. The LTO was then rinsed twice using DMC to remove residual electrolyte, followed by drying under vacuum in the glovebox prechamber for 45–60 min.

For ssNMR measurements, 8–12 mg of the cycled LTO powder was packed in the glovebox inside 2.5 mm zirconia rotors. The sealed rotors were then transferred to the ssNMR spectrometer. For exogenous DNP experiments, about 8–10 mg of the cycled LTO powder were wetted by 4–5 μL solution of 16 mM TEKPol (Cortecnet) dissolved in tetrachloroethane (TCE, Sigma-Aldrich), prepared in the glovebox. This resulted in a moist LTO powder that was packed into 3.2 mm sapphire rotors closed with a Teflon plug and zirconia cap. For endogenous DNP measurements the cycled Mn doped LTO powder was directly packed in the 3.2 mm sapphire rotors inside the glovebox, and closed with a Teflon plug and zirconia cap.

### 2.5 X-ray Photoelectron Spectroscopy

PpLTO electrodes were loaded to the XPS instrument via glovebox purged for several hours with N_2_ to prevent air exposure. XPS measurements were carried out with Kratos AXIS ULTRA system using a monochromatic Al Kα X-ray source (*h*ν = 1486.6 eV) at 75 W and detection pass energy of 40 eV. Curve fitting analysis was based on linear or Shirley background subtraction and application of Gaussian–Lorentzian line shapes.

For depth profiling of the samples an argon gas ion source (Ar^+^-GIS) integrated into Kratos system was applied. With the source high tension (HT) of 3.8 keV and the extractor currents of 25 and 50 μA, the sputtering (removal) rates are in the order of 0.3 and 1 Å/s, respectively.

### 2.6 ssNMR Experiments

Solid-state NMR experiments were performed on 9.4 T Bruker AVANCE III and AVANCE Neo 400 MHz wide bore spectrometers. All experiments were performed with MAS of 25 kHz using a Bruker 2.5 mm triple resonance probe. ^23^Na spectra were referenced to NaCl set at 7.2 ppm, ^1^H and ^13^C spectra were referenced to adamantane at 1.8 and 38.5 ppm (for the CH resonance), respectively, and ^19^F and ^7^Li spectra were referenced to LiF at −204 and −1 ppm, respectively. Quantification was done by integrating the resonances using MATLAB and DMfit.^63^

### 2.7 MAS-DNP Experiments

DNP experiments were performed on a Bruker 9.4 T AVANCE-Neo spectrometer equipped with a sweep coil and a 263 GHz gyrotron system. A 3.2 mm triple and double resonance low-temperature DNP probes were used for the experiments at a spinning rate of 9 kHz. All experiments were performed at about 100 K, with sample temperature of ca. 99 and 105 K without and with microwave irradiation, respectively. All spectra were acquired after the sample temperature was stable. Longitudinal relaxation, *T*_1_, and polarization buildup time with microwave irradiation, *T*_bu_, were measured with the saturation recovery pulse sequence using a train of 50 short pulses separated by 1 ms delays for saturation.

^1^H experiments were acquired using a rotor synchronized Hahn echo sequence. ^1^H–^23^Na cross-polarization (CP) magic-angle spinning experiments (CP-MAS) were performed using a radio frequency (RF) amplitude ramp on the ^1^H channel without ^1^H decoupling. In ^1^H–^13^C CP experiments swept-frequency two-pulse phase modulation ^1^H decoupling was used.^64^^23^Na{^7^Li} and ^23^Na{^19^F} rotational echo double resonance (REDOR)^65^ experiments were implemented in a pseudo two-dimensional manner. In all experiments the signal was first saturated by a train (20–50 repetitions) of short pulses separated by 1 ms delays followed by a relaxation or polarization delay. Exogenous DNP was employed with direct ^23^Na polarization and indirect polarization through ^1^H–^23^Na CP MAS. ^1^H,^23^Na and ^19^F relaxation experiments were analyzed using TOPSPIN and fitted with MATLAB software. ^1^H and ^13^C resonances were referenced to TCE solvent resonances at 6.4 and 74 ppm.

### 2.8 Ab Initio Calculations of ^23^Na NMR Parameters

All Na-containing LTO structures considered here are based on the reported spinel Li_4_Ti_5_O_12_ crystal structure with space group *Fd*-3*m* (ICSD 257309)^66^ and provide possible models for LTO cycled against Na metal in the discharged state. Due to mixed Li/Ti occupancy of the 16d sites, the structure was reduced to its primitive cell and all possible Li/Ti orderings were enumerated in a 1 × 2 × 1 supercell. An Ewald energy algorithm from the Pymatgen library was used to rank those orderings according to their Coulombic interaction energies.^67^ Starting from the lowest energy Li/Ti ordering, 8 symmetrically distinct structures were created by systematically replacing one of the 8 Li atoms in the unit cell with a Na atom, yielding 6 structures with a Na sitting on an 8a tetrahedral site, and 2 structures with Na on a 16d octahedral site. NMR CASTEP^68^ calculations were carried out on these 8 Na-containing structures to determine their respective ^23^Na isotropic chemical shielding constants (σ_iso_). Calculations were conducted using the projector augmented-wave method (GIPAW) and “on-the-fly” ultrasoft pseudopotentials from the CASTEP database.^69,70^ The exchange–correlation term was approximated with the generalized gradient approximation by Perdew, Burke, and Ernzerhof^71^ and relativistic effects were accounted for using the scalar-relativistic zeroth-order regular approximation.^72^ Structures were geometrically relaxed using the Broyden–Fletcher–Goldfarb–Shanno (BFGS) algorithm without any constraints placed on the lattice parameters, symmetry, or atomic positions before the NMR parameter calculations, using a 80 Ry energy cutoff and 4 × 2 × 4 *k*-point grid. A cutoff energy of 90 Ry and a 4 × 2 × 4 *k*-point grid were used for computing the NMR parameters of each structure. Convergence parameters for each set of calculations are as follows: For single point energy calculations, the convergence tolerance was set to 0.5 meV atom^–1^. Geometry optimization calculations used a 0.02 meV atom^–1^ energy convergence tolerance, a 0.05 eV Å^–1^ maximum ionic force tolerance, a 0.001 Å maximum ionic displacement tolerance, and a 0.1 GPA maximum stress component tolerance. The convergence criterion for calculations of ^23^Na NMR isotropic shifts was set to 0.5 ppm.

Computed σ_iso_ values were converted into experimental chemical shifts (δ_iso_) based on a previously reported ^23^Na calibration curve^73^ with the addition of a few oxide-based phases. The computed and experimental shifts for the oxide compounds can be found in the Supporting Information (Table S1), along with the equation for converting between σ_iso_ and δ_iso_.

## 3 Results and Discussion

### 3.1 Characterization of LTO and Electrochemical Performance

LTO with a cubic spinel structure (*Fd*-3*m* space group) was synthesized by a hydrothermal route resulting in LTO with 90–95% phase purity (as determined by pattern refinement through the XRD JADE software) with minor TiO_2_ and amorphous phase impurities (Figure S1a). SEM images of as-synthesized LTO (Figure S1b) reveal the flake-like morphology of the particles, with no significant agglomeration and a particle size ranging from 200 to 300 nm.

**Figure 1:**
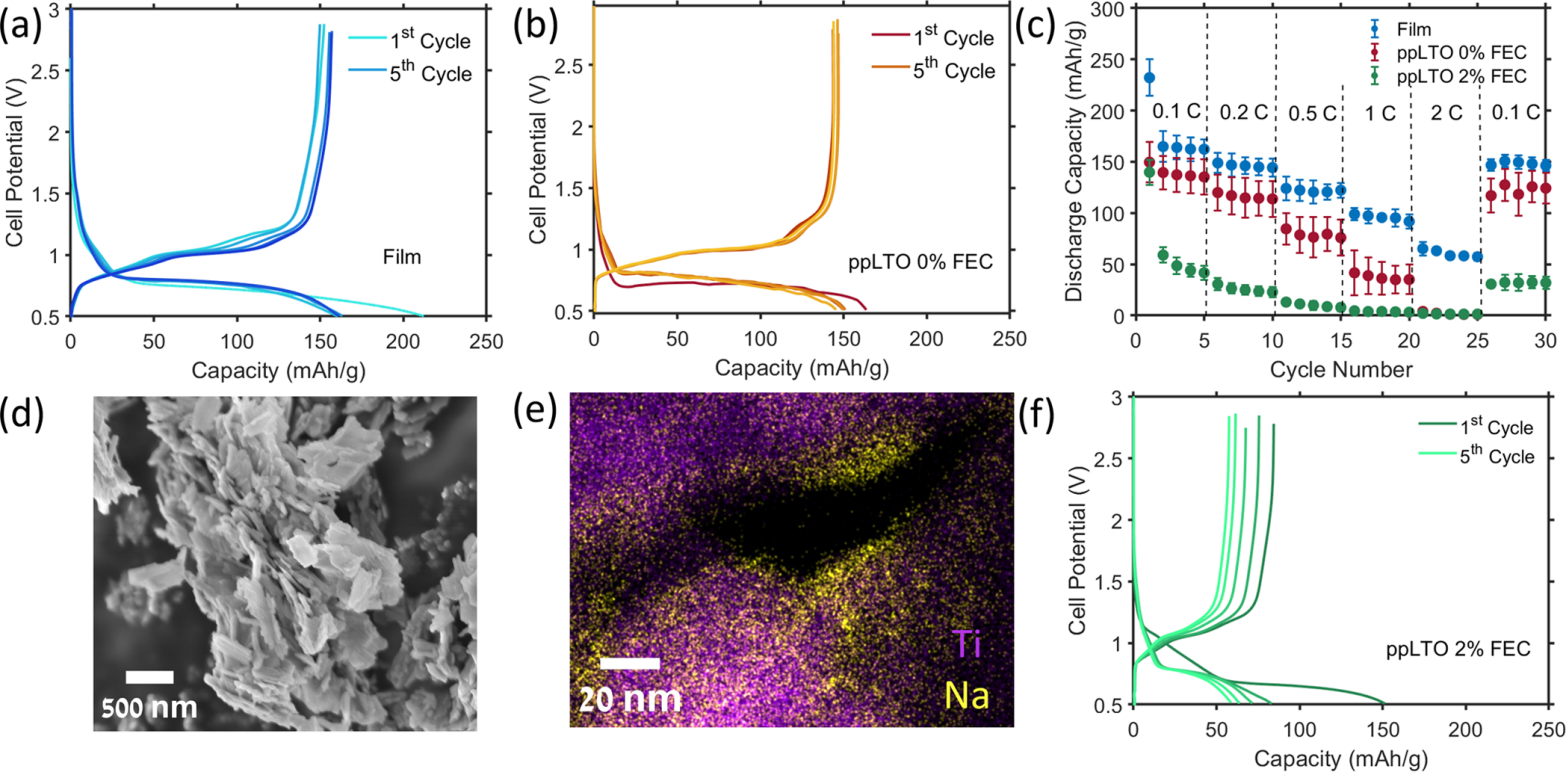
Charge–discharge curves of LTO vs Na metal cells prepared with (a) conventional film electrodes (LTO, carbon black and binder) and (b) pressed powder (pp) LTO cycled with 0% FEC, and (f) ppLTO cycled with 2% FEC. All batteries were cycled at C/10. (c) Discharge capacities as a function of cycle number tested at variable rates for film and pressed powder electrodes cycled with 0 and 2% FEC. (d) SEM image of ppLTO after cycling taken with 5 kV accelerating voltage. (e) TEM-EDS image of ppLTO taken after 20 cycles.

The resulting LTO powder was tested in battery cells in a half-cell configuration where LTO is cycled vs Na metal. According to Huang et al.^51^ electrochemical sodiation of LTO proceeds according to
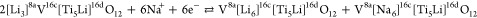
where *V* stands for vacancy and 8a, 16c/d are the Wyckoff indices of atomic positions (tetrahedral and octahedral, respectively) in the *Fd*-3*m* space group. That is, Na ions intercalate into the 16c octahedral sites of the original LTO spinel structure and form a Na-rich phase with a rock salt structure. Due to Na insertion, the Li ions in the 8a sites are pushed to nearby 16c sites due to Coulombic repulsion, leaving behind vacant 8a sites and forming an additional rock salt Li-rich phase. The sodiation of LTO is accompanied by a redox reaction, where Ti(IV) is reduced to Ti(III), and the process is reversed during sodium extraction. The theoretical capacity of LTO is 175 mAh/g, assuming de/insertion of 3 Na^+^ ions per LTO unit. The results of electrochemical cycling of LTO films vs Na metal with NaFSI dissolved in EC/DEC (1:1) as electrolyte are shown in Figure 1a. The first cycle displays a high irreversible capacity, corresponding to SEI formation, and the reversible capacity obtained on subsequent cycles stabilizes at about 86% of the theoretical value. These results are on par with previous reports of nanosized LTO.^74−76^ In the past decade, there have been several attempts to optimize LTO performance in SIBs, primarily centered around particle nanosizing and morphology control, carbon coating and metal doping.^77−81^

Previous studies in our group showed the deleterious effect of carbon black additives, commonly employed to increase the electronic conductivity of the film, on ssNMR sensitivity enhancement via DNP. This was attributed to the absorbance of microwave irradiation by the carbon, resulting in sample heating.^82^ In the case of LTO as a LIB anode, we have shown that good electrochemical performance can still be achieved in carbon-free electrodes by calendaring the films. This formulation enabled significant DNP enhancements in carbon-free LTO following electrochemical cycling.^83^ Here, we opted to use ppLTO as electrode without both the carbon black and the PVDF binder so that the formed SEI could be entirely attributed to electrolyte reduction processes at the surface of LTO. To test our ppLTO electrode formulation, we compared the electrochemical performance of ppLTO and conventional film electrodes (mixed with carbon black C-45 and PVDF), with results shown in Figure 1b. Overall, at the moderate rate of C/10 used here, the voltage profiles are quite similar for both formulations. However, the film-based cells show slightly better capacity retention, as well as improved rate performance (Figure 1c), particularly at high current densities. This is most likely due to the improved electronic conductivity from carbon black and better physical contacts of LTO particles provided by the PVDF binder. Nevertheless, our results suggest that, for SEI studies at C/10, the ppLTO electrodes are comparable to conventional film electrodes with the advantage of enabling high sensitivity ssNMR characterization by leveraging DNP. We note that, in contrast to LTO used in LIB cells, SIB cells are much more sensitive to the LTO morphology. A comparison of the electrochemical performance of different LTO samples in LIB and SIB is provided in the Supporting Information (Figure S2a), indicating the superiority of the hydrothermal route for LTO, most likely due to the high surface to volume ratio obtained through this route. Figure 1d depicts an SEM image obtained on a ppLTO electrode sample after 3 cycles, which shows no significant difference from the pristine LTO electrode (Figure S1b). However, TEM-EDS map obtained on an LTO sample after 20 cycles exhibits a distinct Na-rich surface layer (Figures 1e and S3), which is likely a Na-containing SEI. This is expected based on the significant irreversible capacity loss observed on the first cycle.

Next, we wanted to examine the effect of an electrolyte additive on the electrochemical performance. Due to its reported positive effect on SEI properties,^57,84^ 2% FEC was added to the electrolyte solution. It is well accepted that FEC acts as a sacrificial additive, as its lowest unoccupied molecular orbital is higher than that of the electrolyte, which leads to FEC decomposition before other electrolyte constituents. Figure 1f shows the voltage profile of ppLTO with 2% FEC, exhibiting a high irreversible capacity during the first cycle. Furthermore, the addition of FEC also results in differences in the first cycle voltage profile, as evidenced by an obvious slope between in the 1.2–0.7 V range. This new slope in the voltage profile is in agreement with the sacrificial nature of FEC. Dis/charge curves in subsequent cycles are similar to those obtained with the 0% FEC electrolyte formulation, although with significantly reduced capacity. Surprisingly, in the case of LTO, it is clear that adding 2% FEC has a detrimental effect on the performance. As seen in Figure S2b, the capacity after 20 cycles for ppLTO cells with 2% FEC drops to approximately 50 mA h/g, in contrast to that obtained for cells with 0% FEC, around 140 mA h/g. Figure 1c shows the results from a rate performance test on LTO/Na cells with 0% and 2% FEC, revealing that the addition of 2% FEC causes a severe decrease in the rate performance of the ppLTO electrodes. To verify that these results are not due to the electrode formulation, we performed similar rate performance tests on half cells comprising conventional LTO film electrodes. While overall achieving better performance, the LTO films show a similar trend (Figure S2c), although the difference between 0 and 2% FEC containing electrolyte is milder. As expected, the addition of FEC in the electrolyte results in a high irreversible capacity on the first cycle, suggesting that FEC is reduced to form a different SEI, and the FEC is fully decomposed by the end of the first cycle shown in Figure 1f. Thus, we speculate that the main difference between the two electrochemical systems is the SEI composition and its properties, as will be discussed in the following sections.

### 3.2 SEI Composition

**Figure 2:**
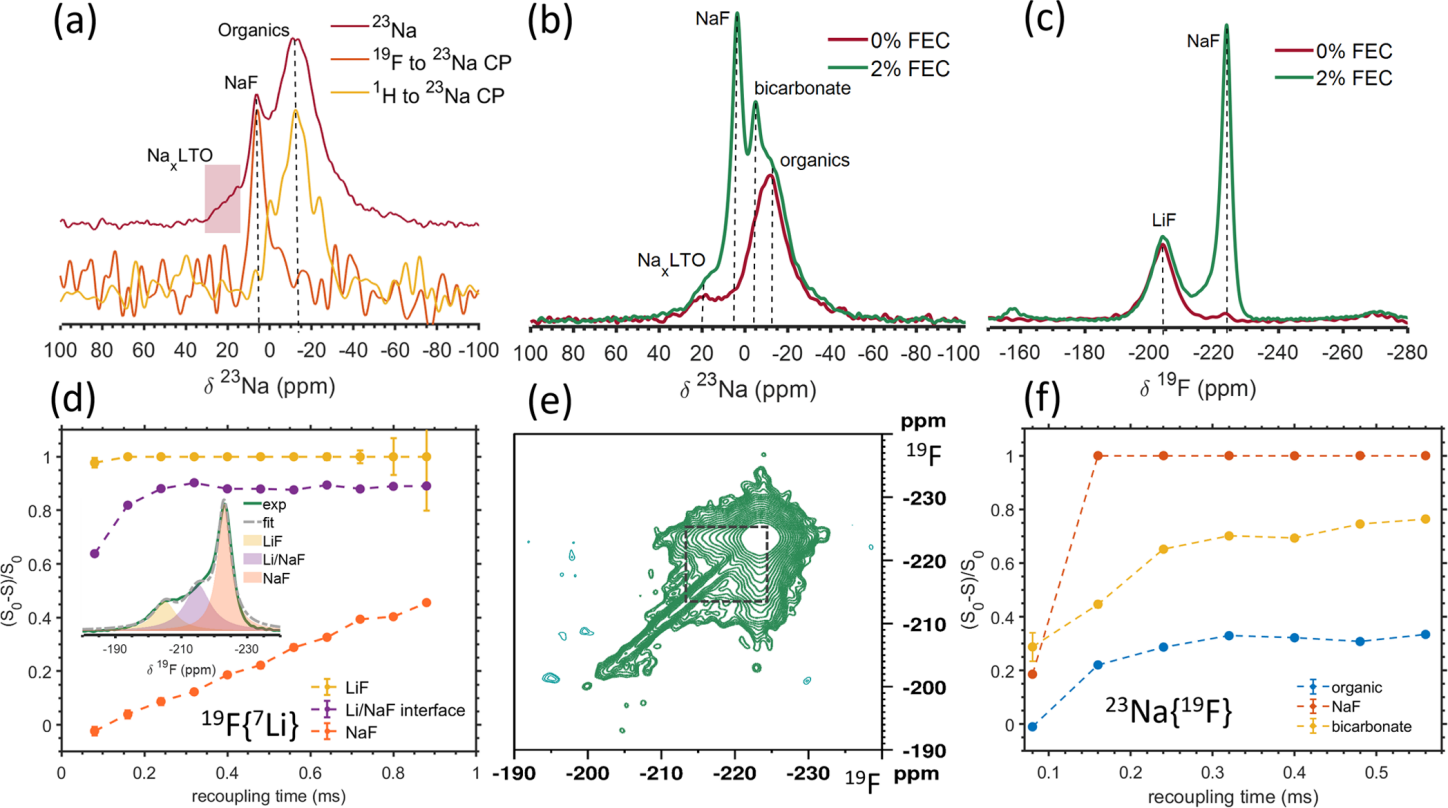
(a) ^23^Na MAS NMR spectrum collected on LTO in the discharged state after 8 cycles with a recycle delay of 2.5 and 256 scans, along with ^19^F–^23^Na and ^1^H–^23^Na CP experiments obtained with a recycle delay, number of scans and contact time of 5.2 s, 3020, 0.25 ms and 2.5 s, 20,480, 1 ms, respectively. (b) ^23^Na MAS NMR spectrum of LTO after 1 cycle with 0 and 2% FEC using recycle delay and number of scans 4, 1024 and 7.5 s, 1024 for the 0 and 2% FEC samples, respectively. (c) ^19^F spectrum of LTO after 1 cycle with 0 and 2% FEC acquired using rotor synchronized Hahn echo with recycle delay of 225 s, 48 scans and 93 s, 128 scans, respectively. (d) ^19^F{^7^Li} REDOR dephasing curve for LTO after 1 cycle with 2% FEC. The REDOR experiment was performed with a recycle delay of 20 s and 48 scans. The inset shows the deconvolution of the spectrum. (e) ^19^F RFDR 2D experiment acquired with 10 ms mixing time, 180 increments, a recycle delay of 19 and 16 scans of LTO after 1 cycle with 2% FEC. (f) ^23^Na{^19^F} REDOR experiment for LTO after 3 cycles with 2% FEC. Recycle delay and number of scans were 5 s, 1664 scans, respectively. All experiments were performed at room temperature and sample spinning at 25 kHz.

Multinuclear NMR was used to gain information on the composition of the native SEI formed on ppLTO electrodes during cycling. The ^23^Na NMR spectrum of LTO in the discharged state after 8 cycles with 0% FEC, shown in Figure 2a, reveals three main ^23^Na environments. To assign the different ^23^Na resonances, CP experiments were performed as they result in polarization transfer between nuclei that are in close spatial proximity. ^19^F to ^23^Na CP indicates that the resonance at 6 ppm corresponds to a Na environment with nearby F, while ^1^H to ^23^Na CP shows that the broad resonance at −10 ppm corresponds to protonated phases. Additional ^23^Na NMR measurements of various reference Na salts are provided in the SI (Figure S4). These spectra further support the assignment of the 6 ppm ^23^Na resonance to NaF, while the resonance at −10 ppm most likely arises from an organic Na salt (NaOH, which would also give rise to a signal in the ^1^H–^23^Na CP spectrum, has a different ^23^Na resonance frequency and line shape). An additional broad ^23^Na resonance is detected at 20 ppm, which corresponds to a Na environment with no fluorine or hydrogen and is not observed in any of the measured salts. We assign this resonance to residual Na within the LTO framework (labeled as Na_*x*_LTO). This assignment is discussed in more detail at the end of this section.

A comparison of the ^23^Na and ^19^F NMR spectra collected on ppLTO cycled with 0 and 2% FEC (Figure 2b,c) shows substantial differences in the SEI composition in these two systems. Adding 2% FEC induces the formation of a considerable amount of NaF, as seen in both the ^23^Na and ^19^F spectra. Moreover, addition of FEC leads to the appearance of an additional resonance between −2 and −4 ppm. This environment was assigned to sodium bicarbonate, NaHCO_3_, based on its ^23^Na resonance frequency^85^ and correlation experiments that will be discussed in the following sections. The formation of LiF, as observed in the ^19^F spectrum in Figure 2c, is not affected by the addition of FEC. Note that the difference in the NaF signal intensity in the spectra obtained on the two 0% FEC samples shown in Figure 2a,b stems from the difference in the number of cycles after which the samples were harvested (8 cycles vs 1 cycle, respectively), consistent with the fact that more NaF forms upon extended cycling. We note these experiments were performed on LTO that undergone varying number of cycles due to the low-efficiency of ^1^H/^19^F–^23^Na CP experiments. CP experiments were feasible on LTO following multiple cycles with thicker SEI that contained overall similar chemical composition to that after 1 cycle.

Insight into the formation of different fluoride phases upon addition of 2% FEC, and their distribution within the SEI, can be gained through correlation experiments. Here, we performed two experiments which provide insight into the spatial proximity between fluoride phases. Namely, ^19^F{^7^Li} and ^23^Na{^19^F} REDOR experiments. Briefly, in an X{Y} REDOR experiment the spectrum of nucleus X is detected following varying number of pulses that are applied on nucleus Y. These pulses lead to decreased signal intensity for nucleus X if it is a few Angstroms away from nucleus Y. The extent of signal loss (so-called dephasing) is then plotted as a function of time, normalized by a reference signal collected with no Y pulses. Such a curve provides qualitative information into the proximity between the chemical environment of X and Y. As the SEI structure is very heterogeneous (and phases are composed of multispin systems) we cannot extract quantitative distance information by fitting the data. Nevertheless, based on previous reports for similar nuclei we expect that a REDOR effect observed in ^19^F{^7^Li} and ^23^Na{^19^F} experiments would indicate nuclear proximity below ∼1 nm.^86^ Furthermore, we performed a ^19^F radio frequency driven recoupling (RFDR) homonuclear correlation experiment. In this two-dimensional experiment, the distance dependent dipolar interaction between ^19^F nuclei is used to identify ^19^F containing chemical environments that are less than ∼2 nm apart.^86^ Such distance results in formation of spectral correlations (in form of cross-peaks) between the different resonances. Figure 2d shows the normalized integral difference of the nondephased (S_0_) and dephased (S) ^19^F{^7^Li} REDOR. The LiF resonance is quickly dephased, as expected since fluorine and lithium are less than 2 Å apart in this phase. The NaF ^19^F resonance slowly dephases, indicating some proximity to ^7^Li environments. An additional broad ^19^F signal centered around −215 ppm, and flanked by the NaF and LiF ^19^F signals, is detected, and its intensity varies across samples (this resonance is more clearly observed in the inset of Figure 2d). This −215 ppm environment is assigned to F species at the interface between LiF/NaF phases, as its signal displays rapid and almost full dephasing within 200 μs, suggesting Li–F distances of less than 10 Å (Figure 2d). The ^19^F RFDR correlation spectrum in Figure 2e shows strong cross peaks between NaF and the Li/NaF resonances. As correlations are expected from F species within less than 2 nm, this indicates nm scale mixing between these two phases, which could also explain the mild dephasing observed for the NaF resonance in the ^19^F{^7^Li} REDOR experiment. Lastly, the ^23^Na{^19^F} REDOR spectrum shown in Figure 2f was performed on ppLTO after 3 cycles with 2% FEC. As expected, the NaF peak dephases most rapidly, followed by the NaHCO_3_ resonance and the resonance assigned to organic phases, the latter showing the least, but non-negligible, dephasing. The ^23^Na{^19^F} REDOR results imply that the organic phases contain significant amount of fluorine, presumably as a result of FEC decomposition. This mechanism is supported by a comparison of ^23^Na{^19^F} REDOR experiments on samples cycled with and without FEC, shown in Figure S5, which reveal a much smaller dephasing of the resonance assigned to organic phases in the absence of FEC.

As the organic Na salts cannot be resolved in the ^23^Na NMR spectrum, we turned to ^13^C detection. The low natural abundance of NMR-active ^13^C (only 1%), compounded by its low NMR sensitivity and the small amount of SEI phases in the sample, prevent the detection of any ^13^C signal from the samples of interest even with ^1^H–^13^C CP. Thus, to enable detection of the organic components of the SEI we employed exogenous DNP. Here, the polarization agents were nitroxide biradicals (TEKPol), which were introduced by gently wetting the LTO powder with a radical solution containing 16 mM of TEKPol in TCE. The sample was then inserted into the DNP probe and kept at 100 K. CW microwave irradiation resulted in polarization transfer from the radicals to nearby protons in the sample, followed by ^1^H–^13^C CP, which allowed sensitive detection of the ^13^C species in the SEI.

**Figure 3:**
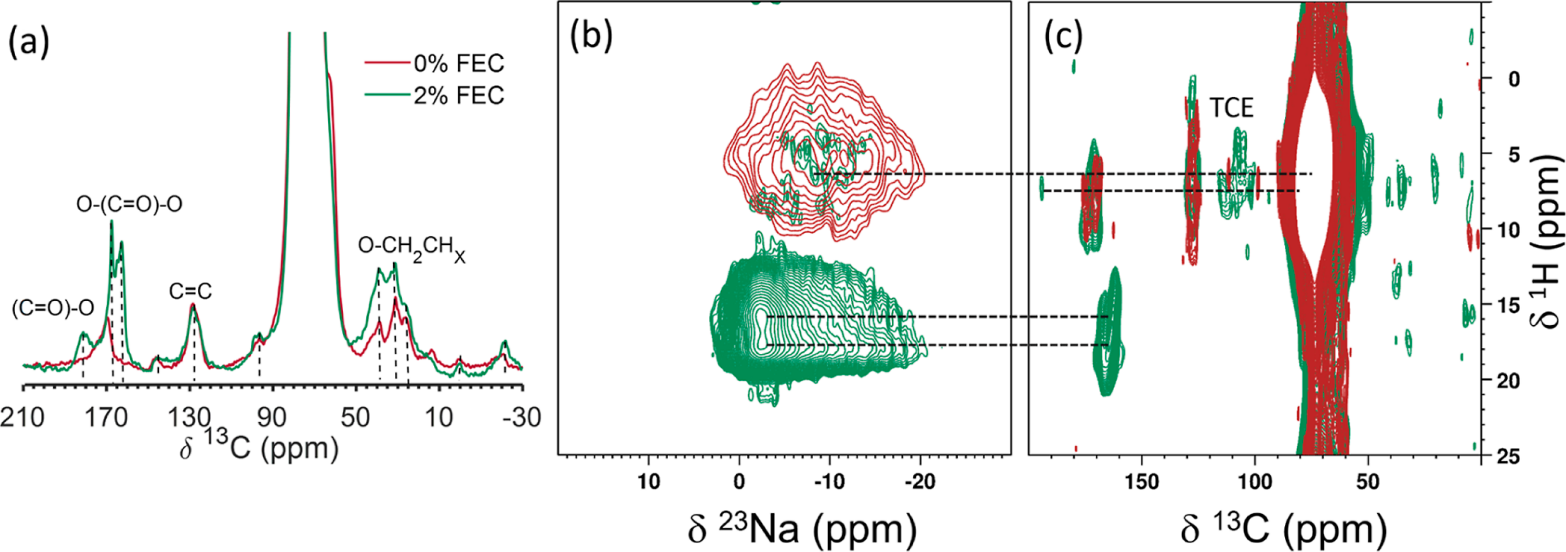
(a) ^1^H–^13^C CP spectra of ppLTO after 1 cycle with 0 and 2% FEC enhanced by exogenous DNP. The spectra were taken using recycle delay, number of scan and contact time of 6 s, 1024, 0.5 ms and 6 s, 984, 2 ms, respectively. 2D heteronuclear correlation (HETCOR) of LTO cycled once with 0 and 2% using CP transfer for correlating (b) ^1^H–^23^Na using 5.5 s recycle delay, 4 scans, 2 ms contact time and (c) ^1^H–^13^C with recycle delay of 5.5 s and 48 scans, contact time of 0.5 and 2 ms for 0 and 2% FEC, respectively. All spectra were acquired with MAS of 9 kHz.

Figure 3a shows the DNP enhanced ^1^H–^13^C CP spectra of LTO after one cycle with 0 and 2% FEC. In both samples, a 64-fold signal enhancement was obtained for the ^1^H TCE resonance transferred to the ^13^C resonances via CP. The spectra were scaled according to the resonance at 74 ppm corresponding to the TCE solvent (truncated to better reveal the resonances corresponding to SEI components), which allows a semiquantitative comparison of the SEI resonances. The resonances at 20–50 ppm are assigned to different alkoxy groups such as CH_3_CH_2_O– or CH_3_O–.^87^ Such environments are associated with the decomposition of the EC and DEC electrolyte solvents. The width of these resonances is due to a distribution of chemical shifts, implying a mixture of disordered products. The addition of 2% FEC to the electrolyte leads to even more pronounced formation of alkoxy species in the SEI. The signal resonating at 130 ppm is assigned to unsaturated carbon species and is attributed to DEC/EC decomposition products; its formation does not seem to be affected by the addition of 2% FEC. The ^13^C resonances at 160–170 ppm belong to carbonate groups such as Na_2_CO_3_ and organic carbonate salts (NaCO_3_R) and are strongly affected by FEC addition. This is corroborated by previous reports indicating that FEC induces the formation of both NaF and Na_2_CO_3_.^47,57^ Additional ^13^C resonances at 163 and 180 ppm appear only in the 2% FEC sample. These resonances are assigned to a bicarbonate and a carboxyl group, respectively.

Additional insight into the organic species found in the SEI could be gained from 2D HETCOR experiments enabled by exogenous DNP. Figure 3c shows an overlay of the 2D ^1^H–^13^C HETCOR DNP spectra obtained on LTO samples after 1 cycle with 0 and 2% FEC. Most of the ^13^C resonances are correlated with the ^1^H resonance at 6.4 ppm, which belongs to the TCE solvent. This suggests that these organic species are accessible to the TCE solvent, meaning that they are found in the outer layers of the SEI, as will be discussed later. However, in the 2% FEC sample, an additional ^1^H resonance is observed at 18 ppm. This 18 ppm ^1^H shift is strongly correlated to the FEC degradation products resonating in the 163–170 ppm range in the ^13^C dimension, suggesting that it corresponds to an acidic bicarbonate species forming upon FEC decomposition. The ^1^H resonance of sodium bicarbonate has previously been reported at 14 ppm, here, it is likely shifted to a higher frequency due to differences in hydrogen bonding within the SEI.^88,89^ Exogenous DNP was also used to detect the ^23^Na species in these phases with the resulting 2D HETCOR spectra shown in Figure 3b for 0 and 2% FEC. In both 0 and 2% FEC, the ^23^Na signal corresponding to the organic phases is enhanced and is correlated with the TCE proton species resonating at 6.4 ppm, indicating that these Na sites are exposed to TCE. In the 2% FEC sample, the ^23^Na resonance associated with NaHCO_3_ at about −4 ppm is enhanced the most. This resonance is strongly correlated to the ^1^H signal at 18 ppm, again suggesting that sodium bicarbonate formation is correlated to FEC decomposition.

Insights from solid-state NMR and exogenous DNP enabled us to gain a compositional map of the native SEI components formed during cycling of LTO, for both organic and inorganic constituents. HETCOR experiments such as REDOR and HETCOR assisted in elucidating the extent of nanoscale-mixing between the different phases of the SEI. It is clear from the results shown above that adding 2% FEC to the electrolyte results in formation of a thicker SEI layer, richer in NaF, as well as containing larger amount of organic species.

### 3.3 Irreversible Na–Li Exchange

**Figure 4:**
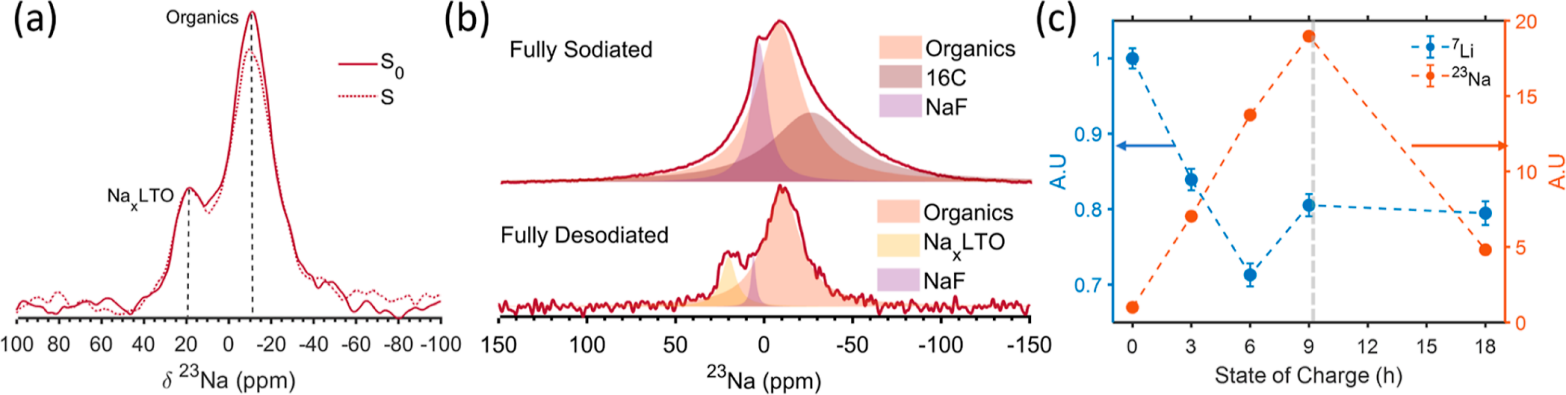
(a) ^23^Na{^19^F} REDOR spectra of an LTO anode after 1 cycle, acquired using recycle delay of 20 s, 128 scans and 1 ms recoupling time with (S) and without (S_0_) pulses on ^19^F. (b) ^23^Na MAS NMR spectra of LTO with 0% FEC after one sodiation and desodiation. The fully desodiated spectrum is the same as in Figure 2b, whereas the full discharge sample was obtained using a relaxation delay of 0.375 s and 5120 scans. (c) Quantification of the ^7^Li and ^23^Na NMR signal of LTO anodes cycled at C/10 and extracted from the cell at different SoC, specified by hours (see Supporting Information for NMR spectra). All experiments were performed with 25 kHz MAS.

We now discuss in more detail the origin of the remaining ^23^Na resonance at 20 ppm (Figure 2a). First, as indicated earlier, this sodium phase does not contain fluorine or hydrogen as it does not appear in the CP spectra (Figure 2a). We also found that this 20 ppm resonance is not in proximity to ^19^F environments, as it does not display a ^23^Na{^19^F} REDOR effect (Figure 4a). This result further confirms that the Na_*x*_LTO is not mixed with SEI components and is not part of the SEI. Furthermore, a comparison of this resonance frequency to that of other ^23^Na-containing reference compounds (Figure S4 and literature reports) suggests that this signal originates from an oxidized Na environment that is different from Na_2_O.^85^ Based on this information, we hypothesize that this resonance corresponds to residual Na ions in the bulk LTO structure at the end of discharge. To test this hypothesis, quantitative ^23^Na and ^7^Li NMR experiments were performed on LTO at different states of charge (SoC, Figure S6a). In Figure 4b, the ^23^Na spectrum of LTO with 0% FEC after one full cycle (fully desodiated) is compared to that of half cycled LTO (fully sodiated). The spectrum collected on fully sodiated LTO contains peaks at −10 and 4 ppm, assigned to organic species and NaF respectively. An additional broad ^23^Na resonance is observed at ca. −40 ppm. As this resonance increases in intensity and shifts toward more negative ppm values with the LTO SoC (Figure S6c–e), it is assigned to Na ions intercalated in the 16c sites. This suggests that the 20 ppm resonance does not correspond to Na intercalated into regular 16c sites. Figure 4c shows the quantification of the ^7^Li and ^23^Na NMR resonances as a function of SoC. In theory, the Li content in LTO should stay constant on sodiation. In practice, however, we observe a clear decrease in Li content upon sodiation to approximately 20% of the theoretical capacity. The decrease in ^7^Li signal, along with the formation of LiF, can be explained by an ion exchange process between the Na ions and the Li ions in the initial LTO anode. This exchange may involve the Na ions present in the electrolyte and occur after the cell is stopped during *ex situ* sample preparation, or could be electrochemically driven and result from the replacement of Li ions by Na ions in the 8a/16d sites of the spinel structure during charge. We note that the drop in ^7^Li signal intensity is not due to LiF formation (which would contribute to the total Li signal measured) but rather a dissolution process of Li ions into the electrolyte. This is supported by the detection of ^7^Li species in solution NMR measurements performed on the electrolyte extracted from a battery cell after cycling (Figure S6f).

To facilitate the assignment of the 20 ppm ^23^Na resonance, first-principles CASTEP calculations were conducted on various LTO-derived structures, with nominal composition Li_3.5_Na_0.5_Ti_5_O_12_ (replacement of 1 Li by Na in a 1 × 2 × 1 supercell of Li_4_Ti_5_O_12_), to determine the chemical shift of ^23^Na species after exchanging with Li into the 8a or 16d sites. The computed ^23^Na chemical shifts are provided in Table 1 and support the assignment of the 20 ppm resonance to Na in the bulk LTO structure, either in an 8a or a 16d site, which appear to be close in frequency. Further details on the calculation of these NMR parameters including quadrupolar parameters of Na positions in LTO are provided in the methods section and Tables S1 and S2. As Li ions are found to leach out of the electrodes, this suggests that Li–Na ion exchange occurs at the periphery of the LTO anode material particles, at the electrode–electrolyte interface. Whether the formation of a bulk Na_*x*_LTO phase is beneficial for the function of LTO as a Na-ion anode is yet to be determined and beyond the scope of this work.

**Table 1:** ^23^Na Chemical Shift Computed by DFT for Na/Li Exchanged on 8a and 16d Crystallographic Sites

crystallographic position of Na in LTO	computed δ (^23^Na) [ppm]
8a	26.7, 26.8, 26.9, 30.7, 30.8
16d	16.1

### 3.4 SEI Structure

It is well accepted that the SEI composition is not the only factor affecting SEI functionality. The order in which phases form at the electrode surface has a strong impact on ionic transport across the SEI.^8,90,91^ Furthermore, a nonhomogenous deposition of SEI phases results in nonuniform charge transfer through “hot spots” in the SEI. This can lead to fast capacity fading due to blockage of regions of the electrode material and, in the case of low voltage anodes such as Na metal and HC, nonuniform Na deposition and dendrite formation. Thus, in addition to the SEI composition, the SEI architecture has a strong effect on the cell performance.

Currently, depth profiling using XPS or mass spectrometry are the most common approaches to gain insight into the SEI structure.^6,7^ However, both approaches are limited in terms of their chemical resolution and require aggressive sputtering techniques that can alter the composition of the SEI. NMR offers a nondestructive approach with which to probe the SEI. Furthermore, the availability of different sources of polarization for DNP can be used to derive structural insight, as we have demonstrated in the case of thin coatings acting as an artificial SEI.^43^ We now turn to examine this approach for probing the electrochemically formed SEI on LTO.

#### 3.4.1 Exogenous DNP

**Figure 5:**
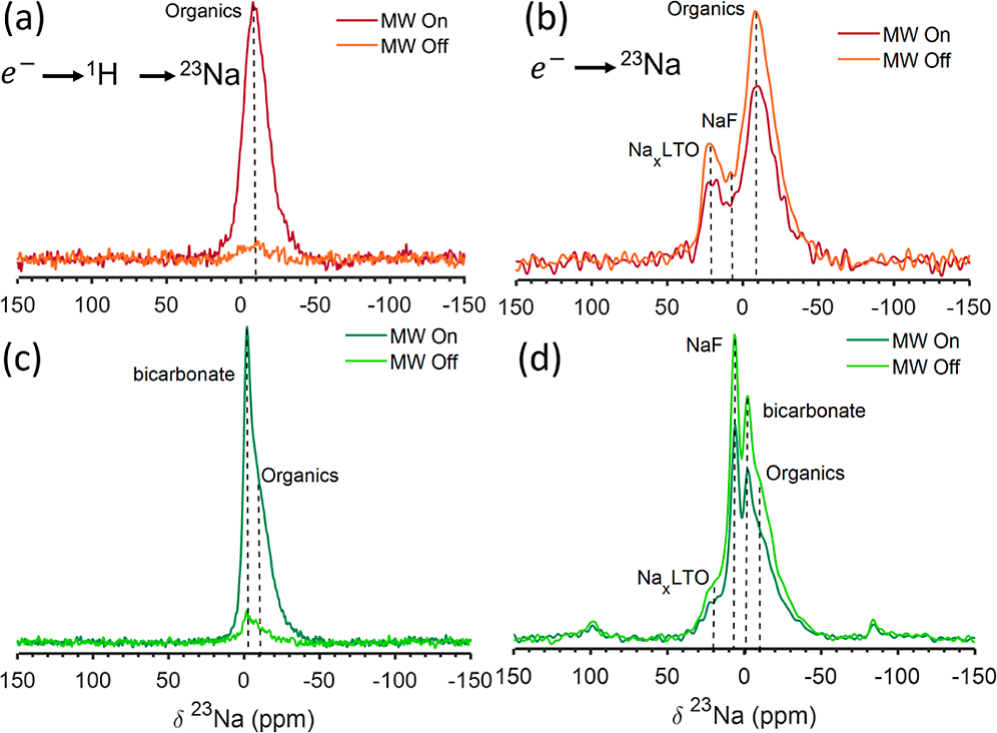
MW on/off spectra of indirect ^1^H–^23^Na CP enhanced by exogenous DNP for LTO after one cycle with (a) 0% FEC and (c) 2% FEC. (a) and (c) were obtained with 6.35 s polarization time, 8 scans and 2 ms contact time. (b,d) are ^23^Na direct excitation exogenous DNP comparing MW on/off spectra of LTO after one cycle with 0 and 2% FEC, respectively. (b) Was acquired using a recycle delay 600 s and 8 scans and (d) using a recycle delay 60 s and 16 scans. All experiments were performed at 100 K and MAS of 9 kHz.

External polarization agents were used in the DNP SENS approach to polarize the SEI by two paths. First, the radicals were used to polarize ^1^H nuclei in the sample (in the solvent and SEI) which gave rise to an enhancement factor of 64 (Figure S7a,b) for both 0 and 2% FEC samples. The ^1^H spectrum is dominated by the solvent resonance at 6.4 ppm and thus is not very informative on the SEI. The ^1^H polarization was transferred to ^13^C (Figure 3a), revealing the composition of the organic phases in the SEI. ^1^H polarization could also be transferred to ^23^Na through CP (Figure 5a,c), resulting in a significant enhancement factor for the nonresolved ^23^Na resonances centered around −10 ppm, which we previously assigned to Na-containing organic species, as discussed in Section 3.2. In the 2% FEC sample, the signal at −4 ppm was enhanced as well, even more so than the −10 ppm resonance (Table S3). Thus, as expected, ^1^H–^23^Na CP provides an efficient path for highlighting the organic SEI components.

Another route for enhancing resonances in the SEI is to transfer polarization from the radical directly to the ^23^Na nuclei at the surface of the LTO particles, as shown in Figure 5b,d. In this case, DNP did not result in increased signal, and on the contrary the ^23^Na resonances decreased in intensity, most likely due to sample heating by the microwaves. Surprisingly, even the organic Na species were not enhanced under these conditions. This can be rationalized considering the very short ^23^Na nuclear relaxation time of these species, which was found to be about 100 ms even at 100 K, as determined by a selective T_1_ measurement following ^1^H–^23^Na CP (Figure S7c). Such short relaxation is likely a limiting factor in direct polarization transfer from the radicals. We note that the ^23^Na T_1_ of the same resonance measured without CP is of the order of 1 s, suggesting that not all of the organic SEI is enhanced via indirect DNP with ^1^H CP. Thus, exogenous DNP provides ssNMR signal enhancement that is limited to the organic phases in the outer layers of the SEI. This effect was similar for LTO samples cycled with and without FEC. In order to probe the inner buried layers of the SEI, endogenous DNP must be employed.

#### 3.4.2 Endogenous DNP

**Figure 6:**
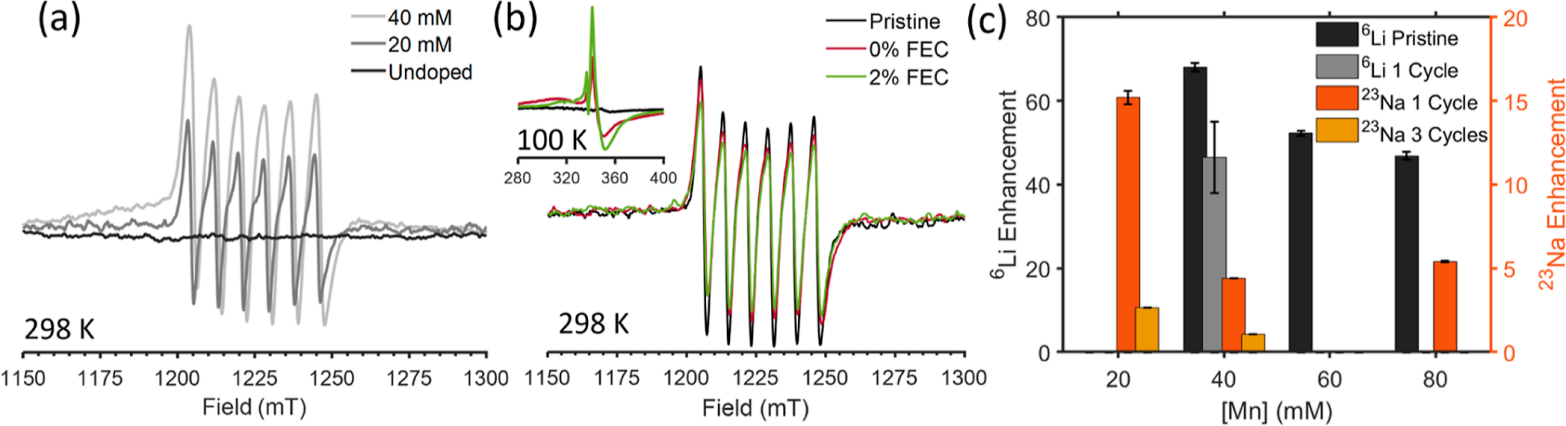
(a) CW-EPR spectra of LTO doped with different Mn(II) concentrations measured on a Q-band spectrometer at 298 K. (b) Q-band CW-EPR spectra of Mn(II) doped LTO before and after cycling with 0 and 2% FEC taken at 298 K. Inset: X-band CW-EPR spectra of Mn(II) doped LTO before and after cycling with 0 and 2% FEC acquired at 100 K. (c) Endogenous DNP-NMR enhancement factors of ^6^Li and ^23^Na of LTO doped with varying amount of Mn(II).

Optimization of Mn(II) dopants for DNP: For endogenous DNP Mn(II) dopants were introduced into the LTO lattice. To this end, Mn(NO_3_)_2_ used as the Mn(II) precursor was added to the hydrothermal synthesis route in varying amounts. EPR measurements were performed in order to confirm the Mn(II) doping of the LTO anode material, with spectra acquired on a Q-band spectrometer are shown in Figure 6a. While the undoped material has no EPR signal, the addition of Mn(II) leads to the appearance of the characteristic six hyperfine transitions of Mn(II) in the EPR spectrum. The increase in signal intensity with Mn(II) concentration clearly indicates successful incorporation of Mn(II) into the LTO anode material.

An important consideration when incorporating Mn(II) dopants into battery materials for endogenous DNP is whether they affect and/or are affected by the electrochemical processes in LTO. For example, the oxidation state of the doped Mn(II) metal ions might change during cycling, resulting in their deactivation for DNP, as was observed for Fe(III) in LTO used as a LIB anode.^83^ EPR measurements of cycled LTO powder were performed to verify that the Mn(II) dopant maintained its oxidation state after cycling. Room temperature spectra acquired at Q-band are shown in Figure 6b, revealing no significant difference between pristine Mn-doped LTO and samples cycled with 0 and 2% FEC, indicating that the Mn(II) species were not affected by the complex electrochemical processes taking place during cycling. The similarity in signal intensity and line-shape also rules out dissolution of Mn(II) ions to the electrolyte (as is often observed in Mn containing cathode materials^92^) or changes in its local environment. However, X-band EPR measurements of cycled LTO acquired at 100 K (Figure 6b, inset) reveal a more complex scenario. These spectra are dominated by a large resonance at ca. 340 mT which is assigned to residual Ti(III) paramagnetic centers that form during sodiation of LTO in the battery cell. The dark blue color of LTO after cycling, shown in Figure S8a,b provides another indication for the presence of Ti(III). These Ti(III) species are visible by EPR only at low temperatures due to their fast relaxation which prevents their detection at room temperature. Ideally, the Ti(III) formed upon sodiation should be fully oxidized back to Ti(IV) by the end of the charging step. However, our EPR results suggest that a significant amount of Ti(III) is still present at the end of the cycling process, likely caused by partial irreversibility of Na intercalation and SEI formation. The amount of Ti(III), based on the EPR spectra, correlates well with the poor electrochemical performance observed for the 2% FEC samples, which have larger irreversible capacity compared to 0% FEC samples. Interestingly, as seen in Figure S8c, the Ti(III) EPR signal decreases with time, indicating a self-oxidation mechanism, possibly by spontaneous reduction of the SEI (note that these samples were sealed in capillaries and stored in an argon glovebox between measurements). The self-oxidation mechanism is also evident by a slow change in color of the LTO sample, from dark blue (characteristic for Ti(III)) to white (Figure S8a,b). Importantly, Mn(II) doping did not have an observable effect on the electrochemical performance of LTO. This is likely due to the very small quantities of Mn(II) dopants introduced for DNP (less than 0.5% in stoichiometry). Thus, EPR characterization confirms Mn(II) incorporation within the LTO lattice and its persistent oxidation state following cycling, a fundamental requirement for it acting as a viable polarization agent for DNP. EPR also revealed significant residual Ti(III) in the samples, reflecting the partial reversibility of the LTO sodiation, which may affect the DNP process as will be discussed below.

Next, we optimized the concentration of the Mn(II) dopant to maximize signal enhancement. Figure 6c shows the ^6^Li and ^23^Na enhancement factors obtained for LTO cycled with 0% FEC as a function of Mn(II) concentration. In comparison with our former study,^83^ relatively high enhancement factors are achieved for ^6^Li after cycling the doped LTO electrodes, with a factor of 50 obtained for a sample doped with 40 mM of Mn(II). This factor corresponds to about 50% of the ^6^Li enhancement obtained in pristine LTO, and is impressive considering the complex electrochemical phase transformation occurring in LTO upon (de)sodiation, as well as the significant contribution from residual Ti(III). We were also able to acquire an ^17^O spectrum via direct polarization from Mn(II) DNP in cycled LTO samples (Figure S8d), demonstrating the efficacy of Mn(II) dopants in polarizing the bulk of LTO. As in our former study on Mn(II)-doped LTO, ^6^Li enhancement increases with decreasing Mn(II) content, seen also in Figure 6c.^38^ This trend can be attributed to the increase in Mn(II) interactions with increasing concentration, which leads to faster Mn(II) electron relaxation and thus lower efficiency in DNP. ^23^Na enhancement shows a similar trend to ^6^Li with higher signal enhancement obtained at the lowest Mn(II) concentration. Further cycling of LTO significantly reduced the ^23^Na enhancement factors, as seen in Figure 6c for samples after 1 and 3 cycles. The reduced ^23^Na enhancement after three cycles is most likely due to higher content of Ti(III) that slightly increases after each cycle due to partial irreversibility of the electrochemical process. Subsequent endogenous DNP experiments were performed on LTO samples doped with 20 and 40 mM Mn(II).

**Figure 7:**
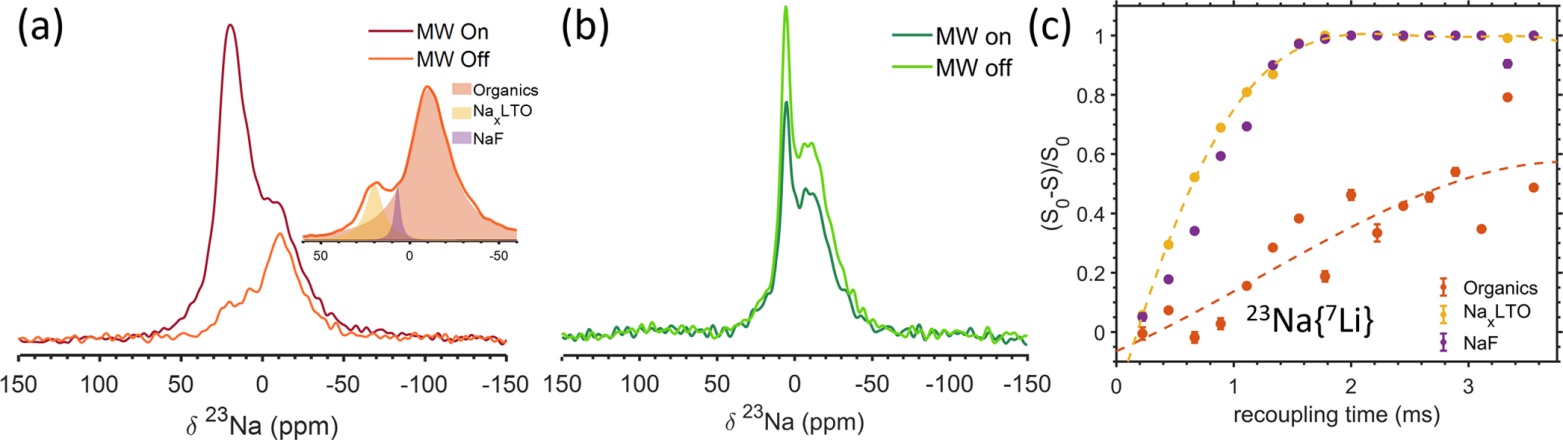
Endogenous DNP-NMR ^23^Na MW on/off spectra for 40 mM Mn(II) doped LTO with (a) 0 and (b) 2% FEC. Experiments were performed with a recycle delay of 1200 and 240 s, respectively, and 4 scans. Inset shows the deconvolution of the MW off spectrum. (c) ^23^Na{^7^Li} REDOR dephasing curve acquired for LTO after 1 cycle with 0% FEC and 20 mM Mn(II) doping. The REDOR experiment was enabled by endogenous DNP with a recycle delay of 140 s and 16 scans. All data acquired at 100 K and MAS of 9 kHz.

Surface enhancement through endogenous DNP: MIDNP from Mn(II) dopants was then used to study the inner layers of the SEI. This was performed through direct polarization of ^23^Na in LTO samples that went through one electrochemical cycle with an electrolyte with and without FEC additive (Figure 7a,b). First, in the samples that did not contain any FEC, we observed significant signal enhancement for all three environments, as shown in Figure 7a for a sample with 40 mM Mn(II). Interestingly, the resonance at 20 ppm, assigned to Na_*x*_LTO, is enhanced the most by a factor of 16–30, followed by NaF and the organic sodium environments have the lowest enhancement in the range 1.5–2 (we note that the enhancement factors slightly varied across different samples but the trends were similar). The NaF enhancement has the highest error due to limited resolution, for the spectra see Figure S9. The high enhancement obtained for Na_*x*_LTO supports the assignment of this resonance to sodium environments within the LTO particles. The three phases also display varying relaxation times, which affects the extent of their enhancement as will be discussed below. In most samples cycled with FEC, we did not observe enhancement of the SEI phases (Figure 7b). We attribute this to the presence of a significant amount of Ti(III) in these samples (see Figure 6b, inset). We note, however, that most of these samples showed non-negligible ^6^Li signal enhancement in the bulk (Figure S10a,b), suggesting that the residual Ti(III) species are trapped closer to the SEI. However, in one sample cycled with FEC, the SEI environments were enhanced, with similar enhancement factors for different SEI components. This is in contrast to the differential enhancement observed in samples cycled with no FEC (Figure S10c,e). Differences in the enhancement distribution across the SEI with and without FEC could be a result of varying SEI structures, as will be discussed later.

The sensitivity from Mn(II) DNP is sufficient to perform correlation experiments which can provide further insight into the spatial proximity of the different phases. To this end, ^23^Na{^7^Li} REDOR experiments with enhanced sensitivity due to MIDNP were performed to determine the proximity of the different Na phases to the LTO. Figure 7c shows results from REDOR experiments for LTO sample with 20 mM Mn(II) after 1 cycle with 0% FEC. In this experiment, the ^23^Na resonance is dephased due to the effect of dipolar interactions with ^7^Li species. In this case, we observe that the Na_*x*_LTO resonance at 20 ppm and the NaF resonance both reach the value of 1, while the resonance associated with the organic species at −10 ppm does not. The full dephasing observed for the NaF and Na_*x*_LTO Na species suggest that in these environments Na sites are a few Angstroms away from Li sites. For the 20 ppm resonance, this further confirms its assignment to ^23^Na sites within the LTO framework resulting in significant ^23^Na–^7^Li dipolar coupling from multiple nearby ^7^Li. The strong dephasing observed for NaF might be due to its proximity to LiF in SEI or to intimate nanoscale contact with LTO. In contrast, the lower dephasing of the organic sites suggests this phase is not in very intimate contact with ^7^Li containing phases. Some dephasing is observed which could be due to minimal contact with the LTO substrate as well as formation of LiF in the SEI, as was also supported by the dephasing observed in ^23^Na{^19^F} REDOR (Figure 4a).

Depth profiling with XPS: Finally, XPS depth profiling experiments were performed to further support our observations from DNP (Figure S11). In this case, we observed a significant decrease in the carbon content with sputtering time, in agreement with our observation of organic species in the outer layer of the SEI. The Li content is roughly constant across the depth of the SEI, possibly due to distribution of LiF in the SEI. On the other hand, the F and Na contents decrease when moving deeper into the SEI, albeit slower than the C content. Furthermore, the decrease in the F and Na contents is more gradual in samples containing FEC. These results are in qualitative agreement with our NMR-DNP characterization, where the SEI formed in FEC had high F content also in its outer layers. Finally, the Ti signal seems to be growing with sputtering time, which is consistent with the sputtering beam moving deeper into the LTO particles. Nevertheless, we note that significant reduction of the Ti species was observed in these experiments (indicated by the formation of metallic Ti, Figure S11c). Such reactivity makes it challenging to quantify the Ti signal, as well as infer on the exact chemical species in the SEI due to chemical changes that may undergo during the sputtering process. Furthermore, the use of pressed powder samples (rather than flat surfaces) makes it challenging to extract reliable spatial distribution of the different species detected, as the signal is contributed from many particles at once.

## 4 Discussion

The use of different polarization sources in DNP allows us to draw some conclusions about the SEI structure. The extent of polarization transfer with both exogenous and endogenous DNP approaches depends on three main rates: the rate of direct polarization transferred from the electron spins (radicals or metal ions) to the SEI, the rate of spin diffusion between the nuclei across phases, and the nuclear relaxation rate which sets the temporal limit for these processes to occur.

Our exogenous DNP results showed that the radicals’ polarization could only be transferred to ^23^Na via protons, with no enhancement obtained in direct ^23^Na DNP. This provides a strong indication that the organic phases, which contain hydrogens, reside in the outer layers of the SEI. The abundance of ^1^H nuclei in these phases allows polarization from the radicals to propagate effectively, either directly to ^1^H in the SEI or indirectly through ^1^H spin diffusion from the frozen TCE solvent. This is further supported by the strong correlations observed for ^13^C and ^23^Na resonances in the SEI and the ^1^H TCE resonance (Figure 3). We attribute the lack of direct enhancement of ^23^Na to its very short relaxation time in the outer organic environments, which would also prevent spin diffusion from the outer SEI layers to the inner ones. As the inorganic phases detected in the SEI (NaF) have relatively long T_1_ relaxation times (>10 s), we would expect it to be directly polarized by the radicals if it were to reside in the outer layers. Thus, our exogenous DNP results suggest that predominantly organic phases are found in the outer layers, which is corroborated by our carbon depth profiling in XPS.

**Figure 8:**
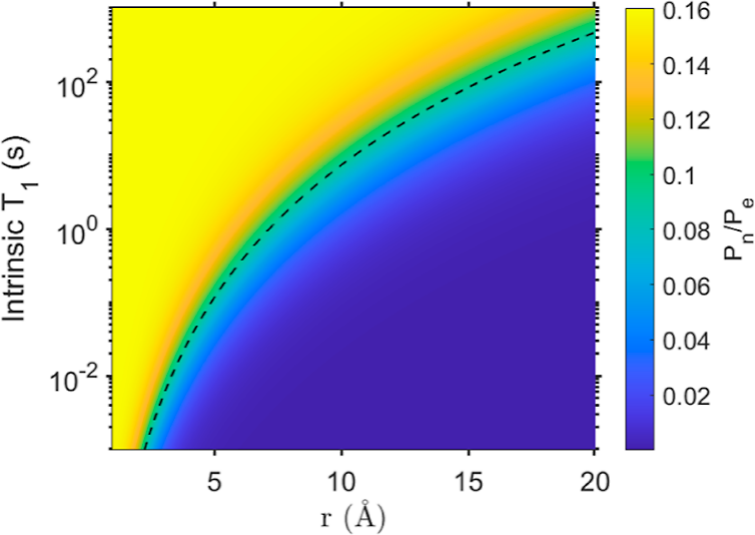
Calculated ratio of ^23^Na polarization (*P*_n_) and electron polarization (*P*_e_) as a function of the dopant-nucleus distance (*r*) and the intrinsic nuclear relaxation time (*T*_1_). The dashed line indicates the distance at which the intrinsic *T*_1_ is equal to the relaxation caused by the paramagnetic ions (PRE). For this calculation an electron relaxation time of 1 μs was used.

**Scheme 2:**
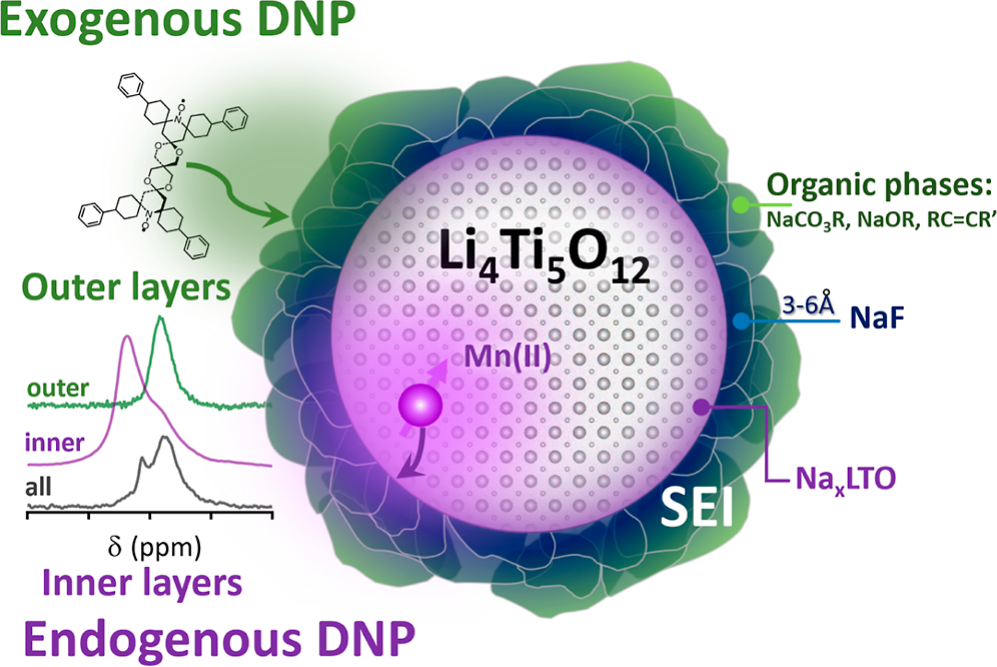
Structural Model of Native SEI Formed in LTO During Cycling Based on Data Acquired with Different Polarization Sources and Correlation Experiments The outer SEI, detected by exogenous DNP, is predominantly organic compounds (green) whereas the inner SEI, detected with endogenous DNP, is more inorganic (dark blue). NaF is the main inorganic species estimated to form a thin layer at the inner SEI. Na_*x*_LTO formed due to Li–Na exchange at the outer layers of the particles is shown in dark purple.

Endogenous DNP enabled enhancement of the inner SEI layers. In this case, we rely on direct polarization of ^23^Na in the SEI. When considering enhancement in the bulk, we have found that in crystalline solids, direct polarization is independent of the nuclear distance from the polarization agent. This is a result of the opposing effects of the paramagnetic metal ions, which act as the main source of nuclear relaxation and of nuclear polarization. Since both nuclear relaxation through paramagnetic agents (paramagnetic relaxation enhancement, PRE) and DNP have the same dependence on the electron–nuclear distance, these two effects cancel each other resulting in efficient polarization transfer across a crystalline lattice.^39^ We have also found that the presence of inherent relaxation sources limits the polarization transfer. In the case of the SEI, we expect a plethora of relaxation processes including ion dynamics, quadrupolar and strong dipolar interactions, as well as presence of paramagnetic defect (such as Ti(III)), all of which provide efficient sources for relaxation. To evaluate the effect of inherent nuclear relaxation on the extent of polarization transfer, the steady state polarization obtained via direct DNP was calculated based on previous derivations.^39^ Here, in addition to relaxation due to paramagnetic dopants, we included inherent relaxation *T*_1n_. The result, shown in Figure 8, suggests that even for an intrinsic relaxation time of 100 s (longer than what was measured for any of the ^23^Na SEI components), the nuclear polarization from DNP drops within 15 Å. For ^23^Na sites with inherent relaxation of 1–10 s (which is closer to the experimental observation for SEI components) direct polarization transfer is limited to 3–6 Å. Thus, we expect that any enhancement obtained from endogenous DNP would only be observed for SEI phases that are deposited directly on the LTO surface. We note that we also assume spin diffusion, which spreads the polarization between nuclear spins, would be inefficient with short intrinsic relaxation times. In this case, the extent of enhancement measured would correspond to the fraction of the phase that is in contact with the LTO substrate compared to its overall fraction in the SEI, i.e. large enhancement corresponds to large fraction on the surface and lower enhancement suggesting only a small fraction of the phase is at the inner SEI. Taking this into account, we conclude that the NaF phase is most likely the first to deposit on the LTO surface followed by the organic components. Considering the discussion above, as well as the quantitative results from ^23^Na MAS NMR revealing that the SEI in this electrolyte is predominantly organic (Figure 2a,b), we propose the following layered model (Scheme 2) for the SEI formed on LTO in NaFSI and EC/DEC electrolyte. In the case of adding FEC to the electrolyte, the result is less conclusive, suggesting a more mixed structure for the SEI. This is also supported by the different REDOR experiments performed, showing significant mixing at the nanoscale between LiF, NaF and the organic SEI (Figure 2).

Our compositional study of the SEI reveals that the SEI formed in NaFSI in EC/DEC electrolyte is composed of NaF, Na_2_CO_3_ and organic carbonates, alkoxy and unsaturated polymer species. The addition of FEC increases the content of organic species in the SEI, specifically alkoxy and carbonate containing phases, as well as results in more pronounced formation of NaF and acidic species such as NaHCO_3_ and carboxylic salts. In terms of its performance, it is clear that FEC, in contrast to the fluoride beneficial effect in LIB cells, decreases the electrochemical performance of LTO as a SIB anode. Cells cycled with the addition of only 2% FEC displayed much higher irreversible capacity, poor capacity retention as well as worse rate performance. Since FEC clearly is consumed in the process, this suggests that the SEI formed with FEC is deleterious to the cell performance. The decrease in rate performance as well as significant amount of residual Ti(III) suggest limited transport of Na^+^ ions, both of which can be due to the formation of ionically insulating SEI, in this case rich in NaF. Thus, we conclude that NaF formation, in this case, is not beneficial in contrast to what is commonly reported for LIB but in agreement with recent reports on potassium-based cells.^93,94^ These results highlight the need to develop new understanding of the SEI formed in SIB, independent of the accumulated knowledge on LIB systems.

## 5 Conclusions

In this work, we showed that NMR can be used as a powerful tool to sensitively characterize the native SEI formed during electrochemical cycling of LTO as part of a SIB. The detrimental effect of the FEC additive on the electrochemical performance in our SIBs was studied and correlated to SEI composition through multinuclear NMR experiments that enabled identification of the various compounds found in the SEI. NMR signal enhancement of ^13^C by exogenous DNP assisted in identifying the organic phases of the SEI which were found to be rich in alkoxy, unsaturated polymers and carbonate species. Furthermore, a sodiated LTO phase (Na_*x*_LTO) that forms due to irreversible Na–Li ion exchange was identified in LTO bulk anode material via quantification of ^7^Li signal during cycling in addition to REDOR experiments and DFT calculations. To the best of our knowledge, this is the first time that such a phase is reported in a sodium LTO cell, however its effect on Na transport is still an open question.

Valuable information regarding the architecture of the SEI was obtained from endogenous and exogenous DNP under the premise that the polarization transfer is distance-dependent. Enhancing the inner SEI layers with endogenous DNP resulted in a ^23^Na spectrum with differential signal enhancement for the SEI components. Analyzing the different enhancement factors revealed that the Na_*x*_LTO phase is found closest to the Mn(II) polarizing agents, followed by inorganic NaF at the inner part of the SEI, and organic species at the outer SEI layer. Signal enhanced ^23^Na{^7^Li} REDOR by endogenous DNP further validated that the sodium found in the Na_*x*_LTO phase is in close spatial proximity to ^7^Li. Performing ^1^H–^23^Na CP with signal enhancement by exogenous DNP confirmed that the organic SEI species are indeed at the outer part of the SEI. By combining the information from both endogenous and exogenous DNP methods, we were able to suggest a model of the SEI formed in LTO due to Na de/insertion including chemical and structural information.

The study of the SEI is extremely challenging due to its sensitive, heterogeneous and disordered nature. The approach presented here has several advantages compared to more traditional approaches for SEI investigations as it benefits from the superior chemical resolution of NMR. Leveraging NMR and the high sensitivity enhancements of DNP, we are able to obtain a detailed chemical compositional map of the SEI. Furthermore, the utilization of multiple polarization sources, demonstrated here for the first time in probing the native SEI, provides spatial information which is used to derive structural models of the SEI. This is a particularly powerful approach as it provides a chemically resolved, nondestructive alternative approach to depth profiling via XPS or TOF-SIMS, both of which have limited applicability in the case of nanoscale SEI layers formed on nonflat substrates. We expect the presented approach, combining exogenous organic radicals and endogenous metal ions, can be extended to other titanate oxides anodes for SIB and K -ion batteries.^94−98^ Furthermore, elucidating both the composition and the distribution of phases across the SEI, and comparison with electrochemical performance, will benefit the development of new electrolyte chemistries. The presented approach, utilizing different polarization sources, can also be envisioned to provide structural insight into the SEI formed on other SIB and LIB anodes, such as Na and Li metal or graphite-based anodes, where the conduction electrons can be utilized as a source of endogenous DNP.^99^ As such, DNP-NMR spectroscopy is a valuable tool in the arsenal of techniques used to probe and understand the SEI, an essential step in the development of improved materials and systems for energy storage.

## Supporting Information Available

Additional materials characterization with XRD, SEM, TEM and electrochemical performance; ^23^Na NMR spectra of reference compounds; NMR quantification of sodium and lithium at different SOC; Details on the CASTEP calculations; ^1^H MAS DNP spectra obtained in exogenous DNP experiments and enhancement factors of different ^23^Na sites obtained in ^1^H–^23^Na CP; Images of the cycled ppLTO electrodes; CW EPR X-band spectra; ^17^O MAS DNP spectrum of Mn doped cycled ppLTO; ^23^Na spectra with deconvolution obtained for various samples with endogenous DNP; XPS depth profiling results (PDF)
