# Supplementary material for: Composition and Structure of the solid electrolyte interphase on Na-Ion Anodes Revealed by Exo- and Endogenous Dynamic Nuclear Polarization—NMR Spectroscopy
Source: J Am Chem Soc. 2024 Aug 22;146(35):24476–92. doi: 10.1021/jacs.4c06823 (PMC11378293; doi:10.1021/jacs.4c06823)
Supplement: Supplementary file 1 — ja4c06823_si_001.pdf [file ja4c06823_si_001.pdf]

Supporting information

# Composition and Structure of the SEI in Na-Ion Anodes Revealed by Exo- and Endogenous Dynamic Nuclear Polarization - NMR Spectroscopy

Yuval Steinberg[1], Elias Sebti[2,3], Ilia B. Moroz[1], Arava Zohar[1], Daniel Jardón-Álvarez[1], Tatyana Bendikov[4], Ayan Maity[1], Raanan Carmieli[4], Raphaële J. Clément [2,3], Michal Leskes[1*]

(1)Department of Molecular Chemistry and Materials Science, Weizmann Institute of Science, Rehovot, 761000, Israel
(2)Materials Department, University of California, Santa Barbara, California 93106, United States
(3)Materials Research Laboratory, University of California, Santa Barbara, California 93106, United States
(4)Department of Chemical Research Support, Weizmann Institute of Science, Rehovot, 761000, Israel
*michal.leskes@weizmann.ac.il

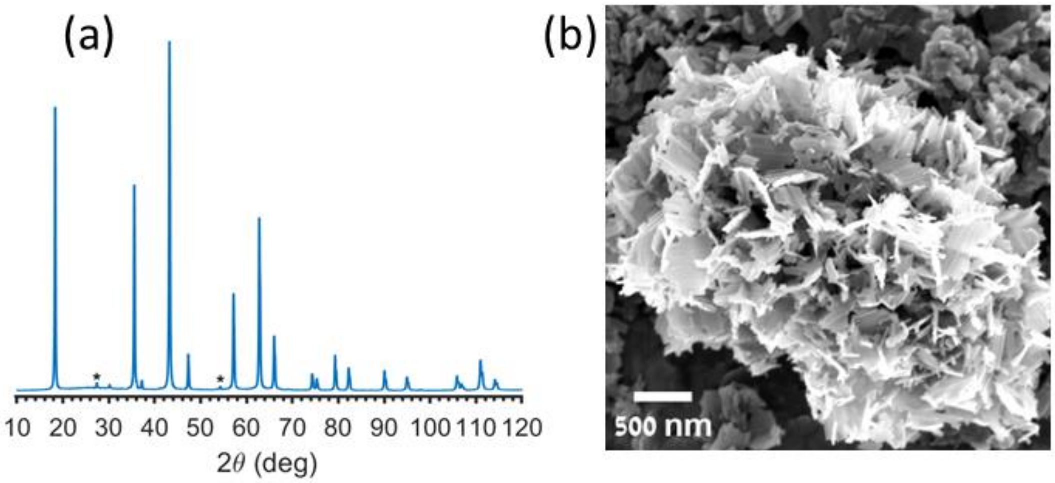


**Figure S1** (a) X-ray diffraction (XRD) pattern of HT-LTO. Reflections from a minor $TiO_2$ impurity are marked with stars. (b) SEM image of the HT-LTO powder acquired prior to electrochemical cycling.

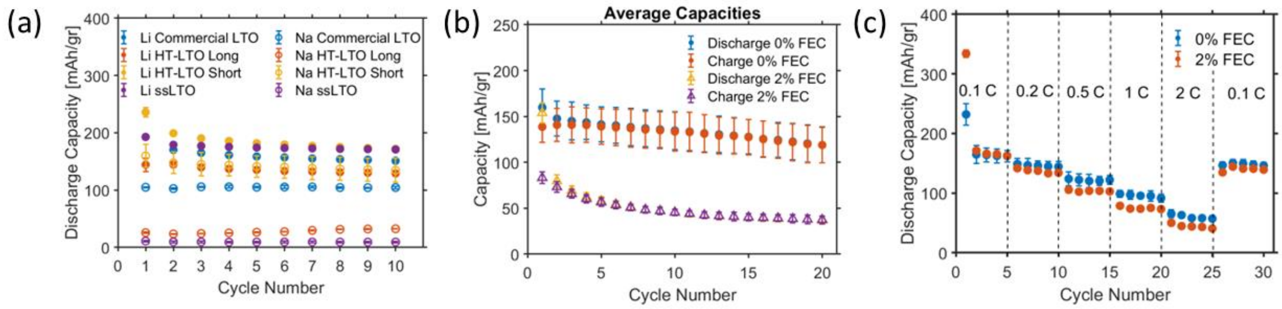


**Figure S2** (a) Comparison of the discharge capacity as a function of cycle number of different LTO powders: commercial, HT synthesis route (HT-LTO) calcined for 2 hours (short) and 7 hours (long), as well as synthesized via a solid-state route (ssLTO). Samples were cycled vs. Li metal (full circles) in 1M $LiPF_6$ in a 1:1 w/w mixture of ethylene carbonate and dimethyl carbonate (LP30) and vs. Na metal (open circles) in 1M NaFSI in 1:1 EC: diethyl carbonate electrolyte. Error bars represent the standard deviation from cycling of several battery cells.
With all LTO samples cycling vs. Li resulted in higher discharge capacity while the capacity obtained when cycling vs. Na metal is much more sensitive to the synthesis route of LTO and thus its surface area and morphology.
(b) Average discharge and charge capacities of ppLTO cycled vs. Na metal, with (triangles) and without (circles) 2% FEC added to the electrolyte.
The addition of FEC results in larger irreversible capacity on the first cycles as well as overall decreased capacity.
(c) Rate performance test of film HT-LTO electrodes cycled vs. Na metal with (orange) and without (blue) FEC in the electrolyte.

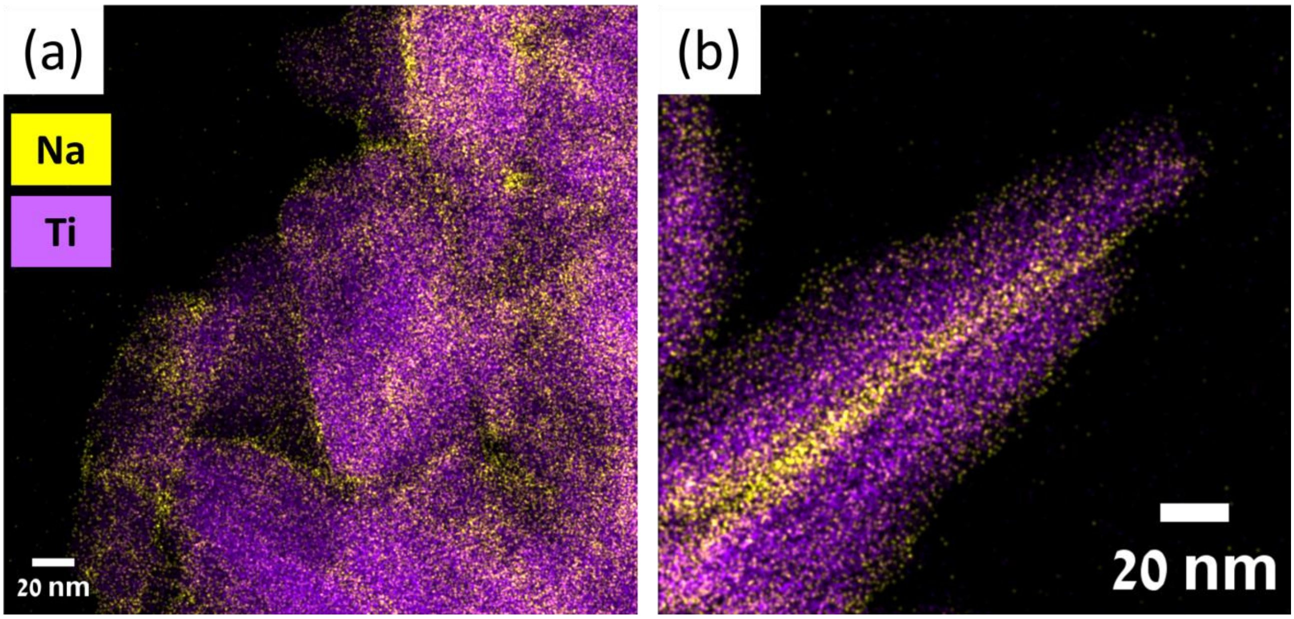


**Figure S3** EDX-TEM images obtained for ppHT-LTO samples cycled without FEC for 20 cycles.

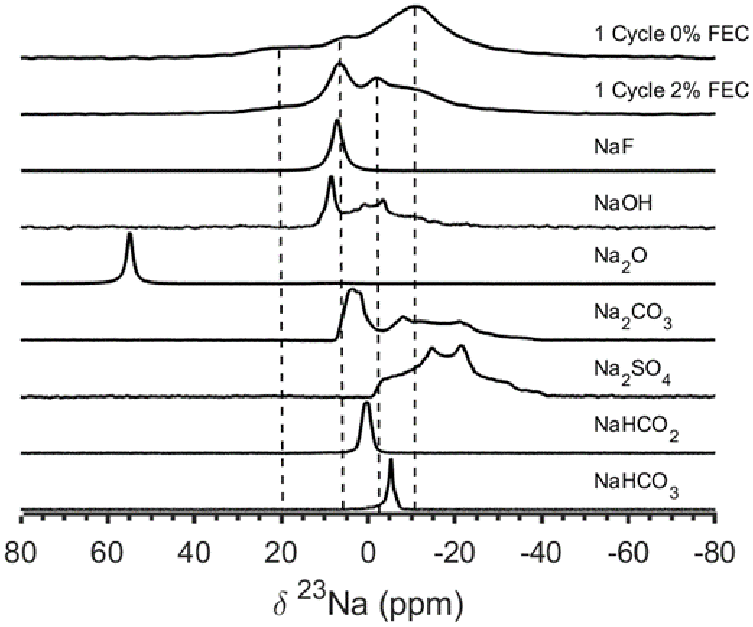


**Figure S4** $^{23}$Na MAS NMR spectra acquired at 9.4 T (400 MHz and 106 MHz $^{1}$H and $^{23}$Na Larmor frequencies, respectively) at 20 kHz MAS frequency. All spectra were acquired with single pulse excitation. Spectra of ppLTO electrodes extracted from cells cycled with and without FEC for 1 cycle are shown on top and compared to a series of spectra acquired for different compounds that are likely to form in the SEI.
Dashed lines are used to guide the eye for the main features in the electrodes' spectra. NaF can be easily identified while the resonance at 20 ppm and -4 ppm do not match any of the measured salts.

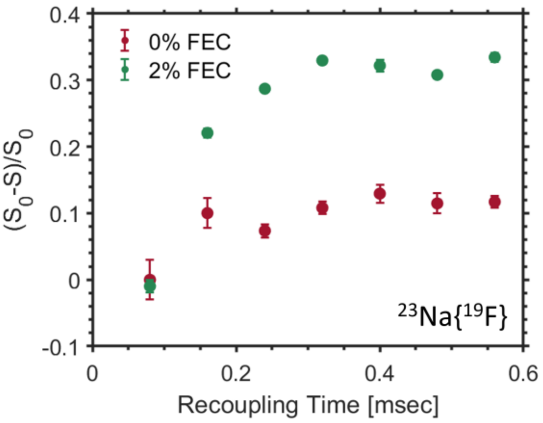


**Figure S5** $^{23}$Na{$^{19}$F} REDOR dephasing curve acquired at 20 kHz MAS for ppLTO cycled with (green) and without (red) FEC in the electrolyte for 3 cycles. The curve is plotted only for the $^{23}$Na resonance of the organic phases in the SEI (-10 ppm).
The organic phases in the SEI have higher proximity to F (or are richer in F content) compared to those formed in the SEI in the absence of FEC.

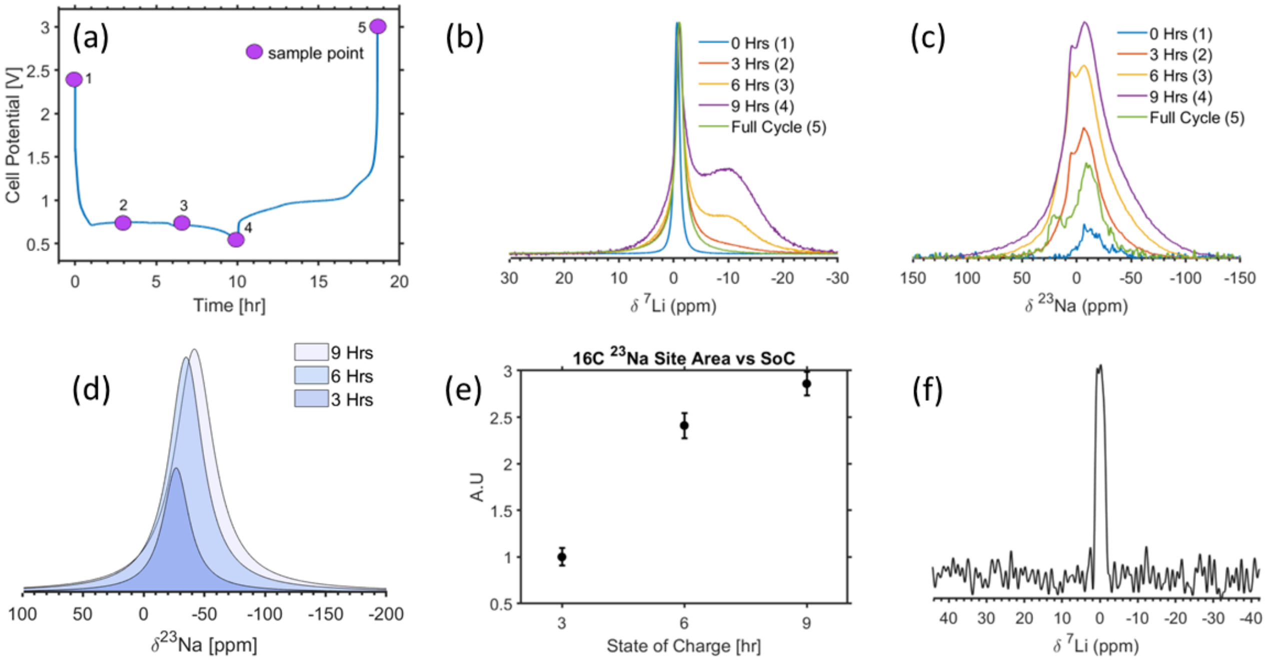


**Figure S6** (a) Voltage profile of the first cycle of HT-LTO vs. cycling time at C/10 (with 10 hours corresponding to full sodiation calculated according to a theoretical capacity of 175 mAh/g). The circles indicate states of charge that were characterized by (b) $^{7}$Li and (c) $^{23}$Na ssNMR and used for quantification of the Li and Na content in the samples. All spectra were acquired on a 9.4 T NMR spectrometer with 2.5mm TR probe, MAS of 20 kHz and using a single pulse excitation. The spectra were acquired with a relaxation delay that was sufficient for all environments to be fully relaxed. (d) Fits of the $^{23}$Na resonance assigned to intercalated Na in the 16c site of LTO (in contrast to SEI) used for the quantification plot shown in (e). (f) $^{7}$Li spectrum acquired on a 11.75 T (500 MHz $^{1}$H Larmor frequency) solution NMR spectrometer for a sample extracted from the separator of the cell stopped at full sodiation. The separator was immersed in water to extract as much of the ions dissolved into the electrolyte during sodiation and the resulting solution was transferred to the NMR tube.

| Compound | CASTEP $\sigma_{iso}$ [ppm] | Exp $\delta_{iso}$ [ppm] | Reference |
|---|---|---|---|
| NaCl | 554.43 | 7.2 | S. F. Dec, G. E. Maciel and J. J. Fitzgerald, J. Am. Chem. Soc., 1990, 112, 9069–9077. |
| $Na_2O$ | 499.49 | 55.1 | G. Kl¨osters and M. Jansen, Solid State Nuclear Magnetic Resonance, 2000, 16, 279–283. |
| $Na_2S$ | 506.83 | 49.7 | G. Mali, M. U. M. Patel, M. Mazaj and R. Dominko, Chem. Eur. J., 2016, 22, 3355–3360. |
| $Na_2S_2$ (Na1) | 550.88 | 8.5 | G. Mali, M. U. M. Patel, M. Mazaj and R. Dominko, Chem. Eur. J., 2016, 22, 3355–3360. |
| $Na_2S_2$ (Na2) | 557.91 | 5.9 | G. Mali, M. U. M. Patel, M. Mazaj and R. Dominko, Chem. Eur. J., 2016, 22, 3355–3360. |
| NaBr | 554.25 | 5.1 | S. F. Dec, G. E. Maciel and J. J. Fitzgerald, J. Am. Chem. Soc., 1990, 112, 9069–9077. |
| NaF | 552.44 | 7.2 | S. F. Dec, G. E. Maciel and J. J. Fitzgerald, J. Am. Chem. Soc., 1990, 112, 9069–9077. |
| NaOH | 537.6 | 21.1 | S. F. Dec, G. E. Maciel and J. J. Fitzgerald, J. Am. Chem. Soc., 1990, 112, 9069–9077. |
| $Na_2SO_4$ | 566.11 | -1.3 | J. Phys. Chem., Vol. 98, No. 6, 1994 |
| $Na_2SiO_3$ | 533.03 | 22.65 | J. Phys. Chem. B, Vol. 105, No. 49, 2001 |
| $Na_3P_30_9$ (Na1) | 574.05 | -7.6 | J. Phys. Chem., Vol. 98, No. 6, 1994 |
| $Na_3P_30_9$ (Na2) | 564.08 | 1.6 | J. Phys. Chem., Vol. 98, No. 6, 1994 |
| $Na_2CrO_4$ (Na1) | 581.73 | -12.8 | J. Phys. Chem., Vol. 98, No. 6, 1994 |
| $Na_2CrO_4$ (Na2) | 572.91 | -6.7 | J. Phys. Chem., Vol. 98, No. 6, 1994 |

**Table S1:** Computed chemical shielding constants and experimental chemical shifts used to obtain the semi-empirical calibration curve for the conversion of $^{23}Na$ CASTEP NMR parameters into values comparable to experiment. The final calibration equation is $\delta_{iso} = -0.8306 * \sigma_{iso} + 468.31$ ($R^2 = 0.9911$).

| Structure | Lattice parameters | Na site | Na position | Computed shift [ppm] | Cq (MHz) |
|---|---|---|---|---|---|
| 8a_Na0 | a, b, c: (5.9806 Å, 13.4349 Å, 5.9806 Å)<br>α, β, γ: (76.860°, 89.628°, 76.860°) | 8a | 0.247, 0.500, 0.753 | 30.7 | -0.545 |
| 8a_Na1 | a, b, c: (5.9688 Å, 13.4968 Å, 5.9714 Å)<br>α, β, γ: (77.633°, 89.957°, 77.012°) | 8a | 0.578, 0.832, 0.0738 | 26.8 | -2.008 |
| 8a_Na2 | a, b, c: (5.9726 Å, 13.5021 Å, 5.9684 Å)<br>α, β, γ: (76.983°, 89.978°, 77.663°) | 8a | 0.928, 0.168, 0.424 | 26.7 | -2.104 |
| 8a_Na3 | a, b, c: (5.9804 Å, 13.4347 Å, 5.9804 Å)<br>α, β, γ: (76.857°, 89.631°, 76.857°) | 8a | 0.00287, 0.000, -0.00287 | 30.8 | -0.564 |
| 8a_Na4 | a, b, c: (5.9726 Å, 13.4973 Å, 5.9673 Å)<br>α, β, γ: (77.006°, 89.959°, 77.633°) | 8a | 0.323, 0.332, 0.327 | 26.9 | -2.049 |
| 8a_Na5 | a, b, c: (5.9670 Å, 13.5039 Å, 5.9715 Å)<br>α, β, γ: (77.626°, 89.947°, 76.952°) | 8a | 0.674, 0.667, 0.677 | 26.9 | -2.046 |
| 16d_Na6 | a, b, c: (6.0092 Å, 13.4342 Å, 5.9447 Å)<br>α, β, γ: (76.899°, 89.906°, 78.131°) | 16d | 0.125, 0.250, 0.875 | 16.1 | 2.825 |
| 16d_Na7 | a, b, c: (5.9447 Å, 13.4327 Å, 6.0094 Å)<br>α, β, γ: (78.122°, 89.919°, 76.907°) | 16d | 0.125, 0.750, 0.875 | 16.1 | 2.813 |

**Table S2:** Details on computed shifts and quadrupolar parameters of Na positions in relaxed Na-containing LTO structures. Further specifications on construction of each crystal structure and calculation methodology can be found in Section 2.8 of the manuscript.

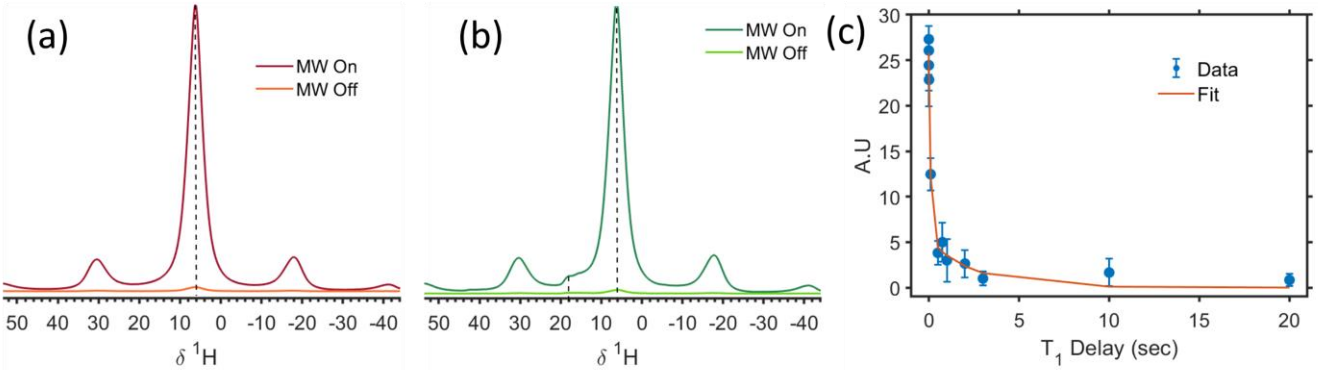


**Figure S7** $^{1}H$ DNP-NMR rotor synchronized Hahn echo spectra acquired at approximately 100 K and 9 kHz MAS. Spectra are compared with (MW on) and without (MW off) microwave irradiation for samples that were wetted with a 16mM TEKpol solution. Two samples are compared (a) ppLTO cycled for 1 cycle without FEC and (b) with FEC. In both cases similar enhancement is obtained for the main resonance observed of the tetrachloroethane (TCE) solvent at 6.4 ppm. The additional resonance observed in the samples cycled with FEC at 18 ppm, assigned to $NaHCO_3$, is also enhanced by the microwaves.
(c) Indirect $^{23}Na$ $T_1$ measurement obtained with $^{1}H$-$^{23}Na$ CP at 100 K with no microwave irradiation for a ppLTO sample cycled for 3 cycles. The resonance contributing to the measurement is the organic phases in the SEI (at -10 ppm) and the curve was fitted with a biexponentially decaying function resulting in a $T_1 = 90 \pm 20$ ms and $T_1 = 2500 \pm 1500$ ms in a 4:1 ratio.

| Sample | Peak | Nucleus | Enhancement |
|---|---|---|---|
| 0% FEC | Organics | $^{23}Na$ | 18 ± 7 |
| 2% FEC | Organics | $^{23}Na$ | 7 ± 2 |
| | $NaHCO_3$ | $^{23}Na$ | 12 ± 4 |

**Table S3** The $^{23}Na$ enhancement factors obtained in indirect ($^{1}H$-$^{23}Na$ CP) exogenous DNP experiments performed on ppLTO samples cycled with and without FEC

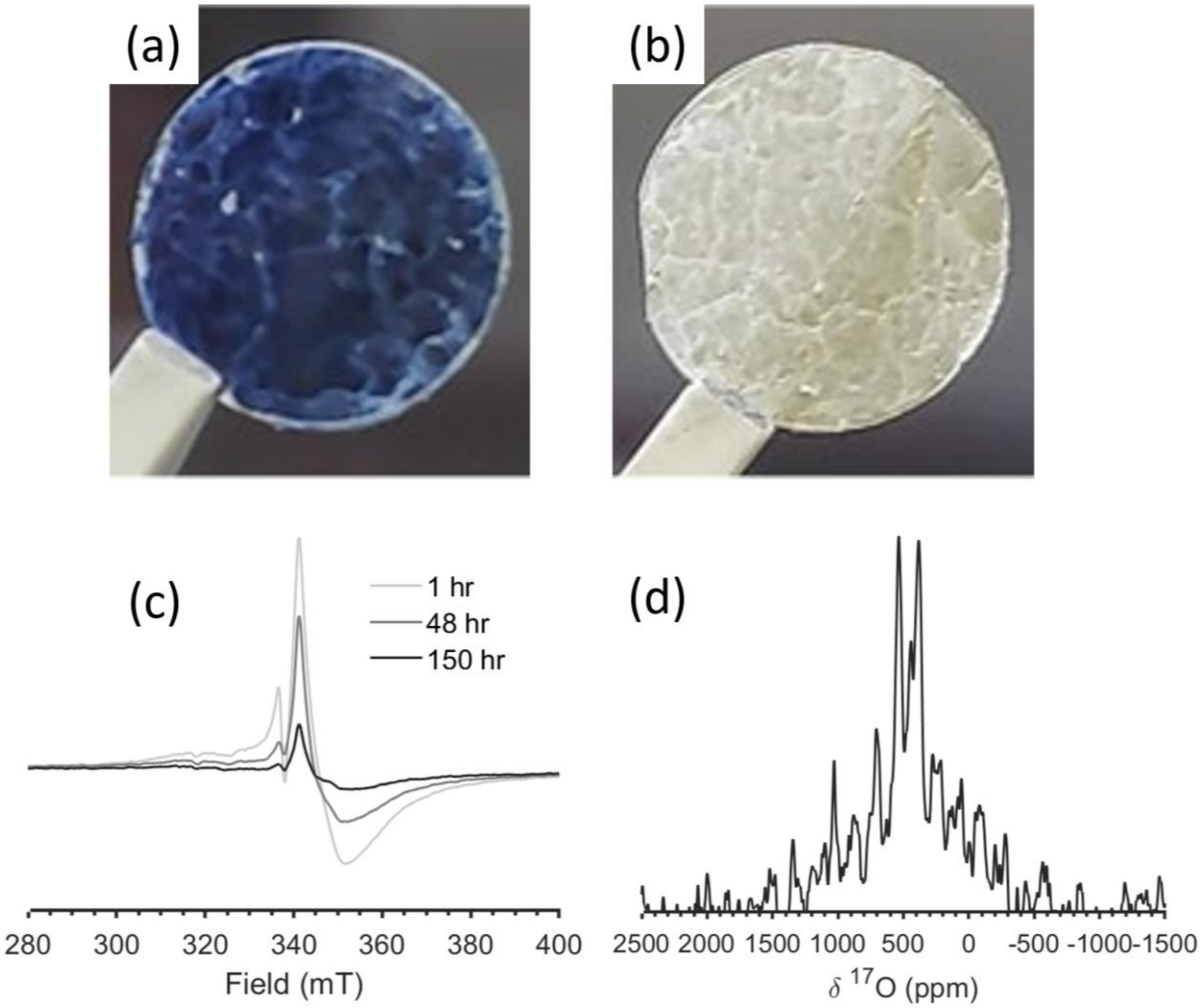


**Figure S8** Photos of a ppLTO electrode (a) right after it was extracted from the cell and (b) after a few days in the glovebox. (c) X-band field sweep EPR spectra acquired at 100 K for cycled ppLTO powder sealed in a quartz capillary in the glove box. Spectra were acquired after 1 hour form extraction from the battery cell, 48 and 150 hours, showing the decrease in EPR signal intensity. (d) $^{17}O$ direct MAS-DNP rotor synchronized Hahn echo spectrum acquired for a 40mM Mn(II) doped ppLTO sample following one cycle. The measurement was acquired with microwave on at the optimal position for positive solid effect enhancement from Mn(II) to $^{17}O$. The spectrum was collected at approximately 100 K, 9 kHz MAS, polarization time of 100 s and 512 scans from a sample weighing 13 mg (following cycling).

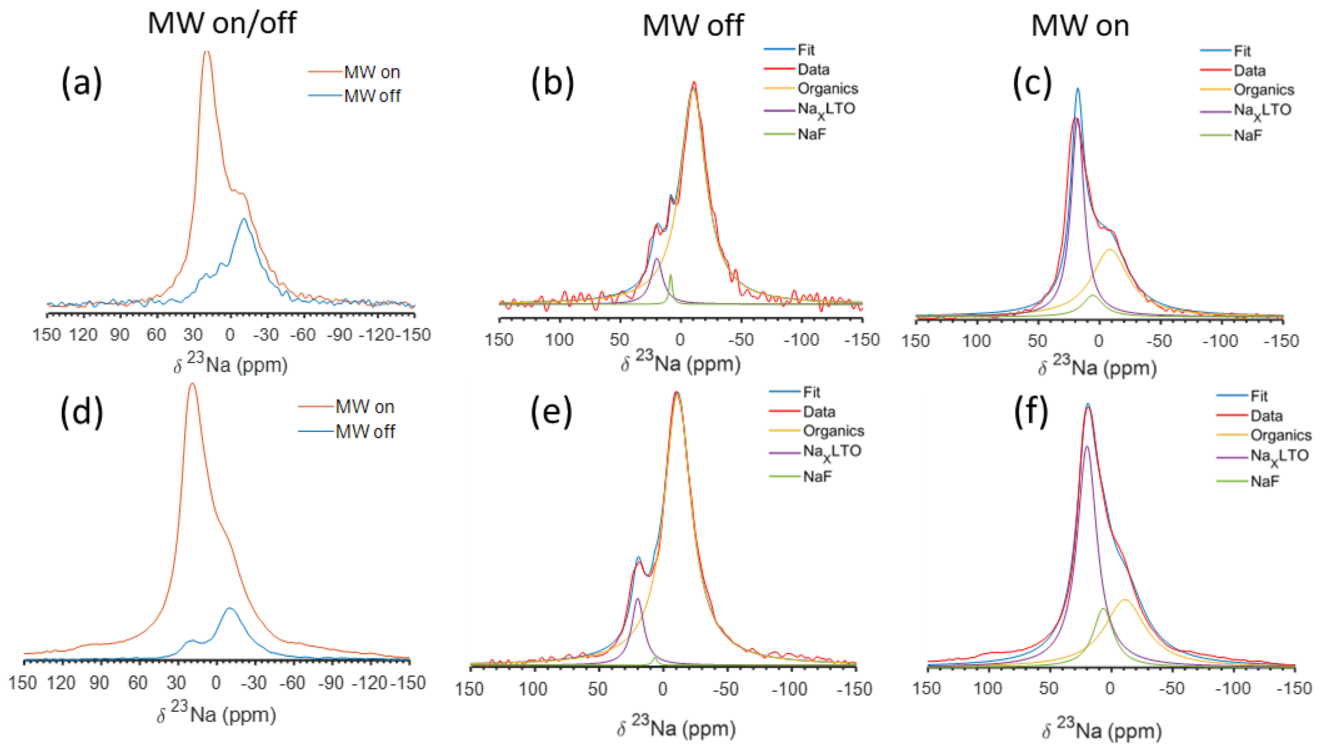


**Figure S9** $^{23}$Na MAS-DNP spectra acquired with direct excitation at 100 K and 9 kHz MAS for 40mM Mn(II) doped ppLTO samples cycled once without FEC. Spectra were acquired with (orange) and without (blue) microwaves. In (a) and (d) the samples weight was 13.1 and 12 mg and the spectrum was acquired with 4 scans and polarization/relaxation delay of 1200 and 1000 s, respectively. The enhancement for each component (given in the following table) was determined by deconvolution of the spectra acquired with and without microwaves shown in plots (b,c) and (e,f).

| S9a-c | Enhancement |
|---|---|
| Organics | 1.45 ± 0.02 |
| NaLTO | 16 ± 2 |
| NaF | 16 ± 3 |
| total | 2.93 ± 0.01 |

| S9e-f | Enhancement |
|---|---|
| Organics | 2.17 ± 0.02 |
| NaLTO | 31.5 ± 0.7 |
| NaF | 199 ± 39 |
| total | 5.88 ± 0.01 |

**Table S4** Enhancement factors for the different environments detected in the spectra presented in Figure S9. The large enhancement factor for NaF in spectrum (d) is likely overestimated due to the difficulty in resolving this resonance in the spectrum acquired with no microwaves.

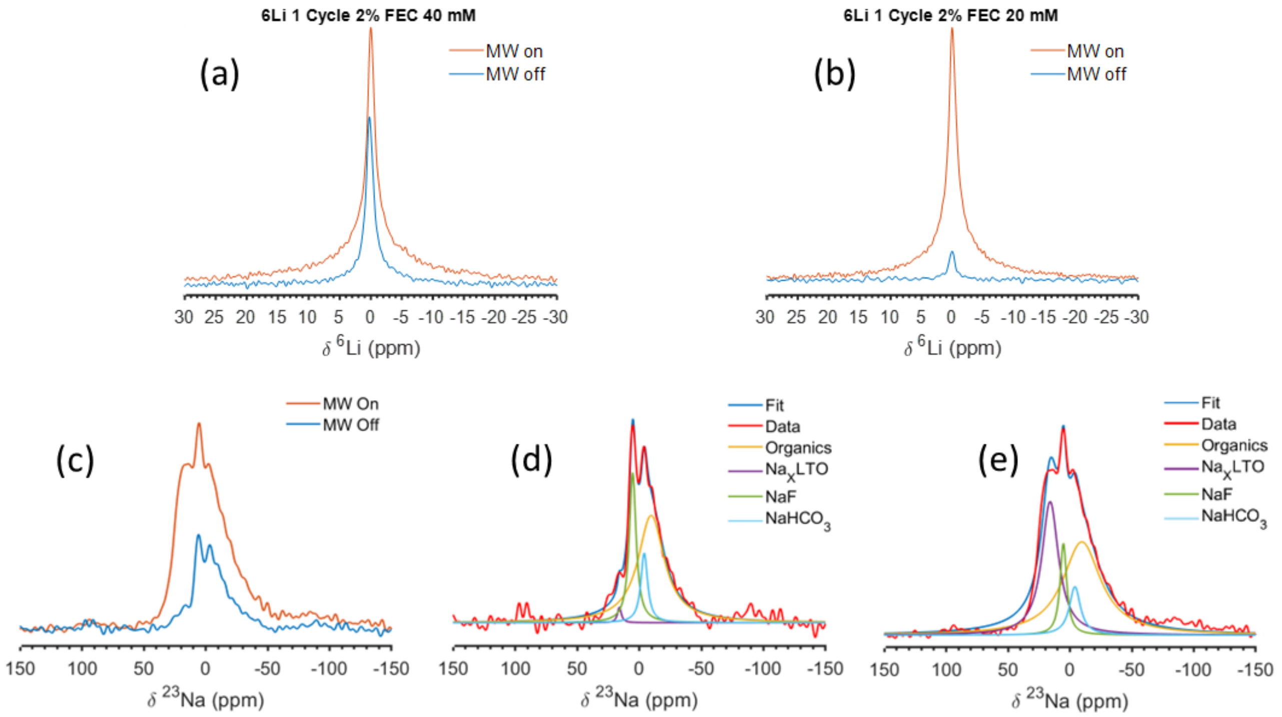


**Figure S10** Single pulse, direct excitation $^{23}Na$ spectra of Mn(II) doped ppLTO samples cycled with 2% FEC in the electrolyte acquired at 100 K and 9 kHz MAS with (orange and without (blue) microwaves. In (a,b) $^{6}Li$ spectra acquired with 10.6 and 3.7 mg of sample, 2 scans and polarization relaxation delay of 500 and 800 s, respectively. (c) $^{23}Na$ spectra for the second sample (10.6 mg)

| S10c-e | With STD in fit |
|---|---|
| Organics | 2.62 ± 0.06 |
| NaLTO | 79 ± 28 |
| NaF | 1.24 ± 0.01 |
| $NaHCO_3$ | 2.37 ± 0.06 |
| total | 3.31 ± 0.05 |

**Table S5** Enhancement factors for the different $^{23}Na$ resonances in Figure S10c, determined from the deconvolution presented in Figure S10d-e.

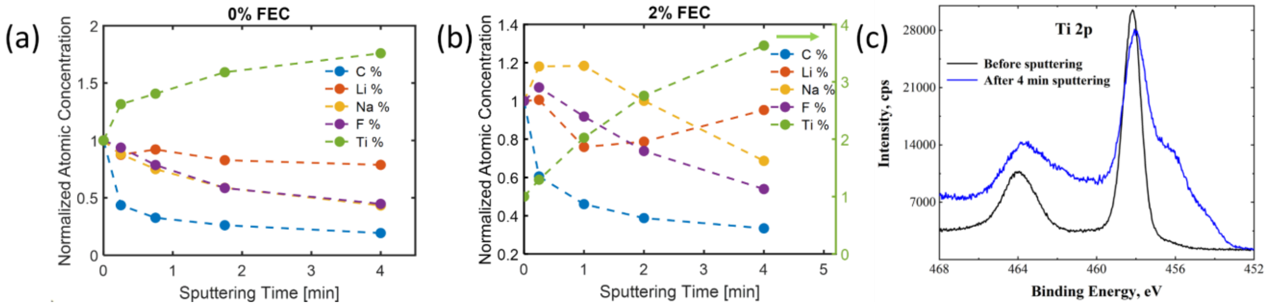


**Figure S11** The contribution in % for different elements as determined by integration of the XPS spectra of ppLTO cycled (a) without and (b) with 2% FEC in the electrolyte and acquired as a function of the sputtering time. (c) Ti 2p XPS spectra before and after 4 minutes of sputtering revealing increased formation of Ti metal (at lower binding energy) suggesting possible reduction of the substrate during sputtering.
